# MetaCell: analysis of single-cell RNA-seq data using *K*-nn graph partitions

**DOI:** 10.1186/s13059-019-1812-2

**Published:** 2019-10-11

**Authors:** Yael Baran, Akhiad Bercovich, Arnau Sebe-Pedros, Yaniv Lubling, Amir Giladi, Elad Chomsky, Zohar Meir, Michael Hoichman, Aviezer Lifshitz, Amos Tanay

**Affiliations:** 1 Department of Computer Science and Applied Mathematics, Weizmann Institute of Science, Rehovot, Israel; 2 Department of Immunology, Weizmann Institute of Science, Rehovot, Israel

**Keywords:** RNA-seq, scRNA-seq, Graph partition, Multinomial distribution, Sampling variance, Clustering, Smoothing

## Abstract

scRNA-seq profiles each represent a highly partial sample of mRNA molecules from a unique cell that can never be resampled, and robust analysis must separate the sampling effect from biological variance. We describe a methodology for partitioning scRNA-seq datasets into *metacells*: disjoint and homogenous groups of profiles that could have been resampled from the same cell. Unlike clustering analysis, our algorithm specializes at obtaining granular as opposed to maximal groups. We show how to use metacells as building blocks for complex quantitative transcriptional maps while avoiding data smoothing. Our algorithms are implemented in the *MetaCell* R/C++ software package.

## Background

Single-cell RNA-seq (scRNA-seq) is used extensively for discovery and identification of cell types, for characterizing transcriptional states within them, and for inference of continuous gene expression gradients linking these states. These phenomenological observations are used for creating cell type atlases and as a starting point for analysis of different cellular processes, including differentiation, cell cycle, and response to stimuli [1–9] (reviewed in [10]). The advent of scRNA-seq increased the resolution of models for transcriptional regulation by orders of magnitude compared to prior bulk methods, allowing precise and unbiased analysis of small cell populations as well as opening the way to quantitative modeling of subtle within-population effects.

As technology matures, the analytical basis for interpreting scRNA-seq experiments must become more principled. In a way similar to other experimental strategies aiming at improved resolution, scRNA-seq relies on the ability to integrate a large number of highly noisy measurements for inferring a high-resolution model of some target sample. In analogy, when performing optimal reconstruction of a microscopic sample, a typical microscopic sensor can reduce noise by resampling the same pixel or voxel, trading instrument time with precision and resolution. In scRNA-seq, the major source of technical noise (not to be confused with various systematic biases) is introduced through partial sampling of some 1000–10,000 RNA-molecules from the pool of RNA within a cell, generating a highly discrete and noisy estimation for the concentration of any RNA species in this cell except very few super-high abundance genes. In contrast to the microscopy analogy, the same cell cannot be revisited and resampled to decrease sampling noise, since scRNA-seq technology involves lysing the cell. Instead, integration of data from different cells must be used to simultaneously capture the true biological variance among cells and the purely technical sampling variance of the experiment.

When scRNA analysis is tuned toward cell type detection [6, 11], the implicit model assumption is that single cells derived from the same transcriptional cluster are approximately identical. In this case, sampling noise can be overcome by pooling the molecules from a sufficiently large number of cells, such that the expected number of sampled transcripts (or unique molecular identifiers (UMIs)) from each significantly expressed gene allows precise inference of the concentration of this RNA species in the idealized cell state that the cluster represents. When aiming at modeling more subtle molecular states, in particular those involving dynamics of cellular differentiation or response to stimuli, the clustering state homogeneity assumption can no longer hold. In these scenarios, current techniques combine handling of sparse data with modeling (implicitly or explicitly) of cellular dynamics [3, 12–24]. Inference of robust cell-to-cell similarity metrics from sparse data is commonly used for construction of *K*-nn graphs over which dynamics are inferred. Smoothing sparse data [25–27] or imputation of transcriptional states [25, 28–30] was proposed as a possible pre-process for modeling similarity in the data. Model-based inference of transcriptional states from sparse data is on the other hand still difficult to derive, since parametric models for single-cell RNA-seq data are lacking. Even though a basic parametric model for the sampling noise in scRNA-seq profiles can be easily assumed, it is not routinely explicitly integrated within a broader context of model inference from scRNA-seq data.

In this paper, we introduce the notion of *metacells* and develop a methodology for inferring and using them. A metacell (abbreviated MC) is in theory a group of scRNA-seq cell profiles that are statistically equivalent to samples derived from the same RNA pool. Such profiles should therefore be distributed multinomially with predictable variance per gene (approximately proportional to the mean) and near zero gene-gene covariance. Moreover, given a set of scRNA-seq profiles that are derived from the same multinomial distribution, it is trivial to infer the model parameters and establish their statistical confidence. If an entire scRNA-seq dataset could be decomposed into disjoint metacells with sufficient coverage per metacell, many difficulties that follow from the sparsity of the data would be circumvented. In practice, one cannot assume a perfect metacell cover of the scRNA-seq dataset a priori, and we found that directly searching for metacells using a parametric approach is highly sensitive to the many intricacies and biases of the data. Instead, we propose to use non-parametric cell-to-cell similarities and partition the resulting *K*-nn similarity graphs into densely connected subgraphs, which are filtered to derive approximately multinomial metacells. Metacells can then serve as building blocks for describing complex gene expression distributions with minimal parametric assumptions, scaling well with the number of cells and providing a more accurate approximation when increasing the number of sampled cells.

We implemented tools for deriving metacells and analyzing scRNA-seq data using them in the new R/C++ package MetaCell. The utility of the approach was recently demonstrated in scenarios involving analysis of mammalian hematopoiesis differentiation [31], immunotherapy [32], blood cancer [33], and inference of cell type decompositions in comparative whole organism scRNA-seq [34, 35]. Here we perform in-depth analysis of the model and its performance through re-analysis of datasets including 8000 and 160,000 peripheral blood mononuclear cells (PBMC), and by dissecting two whole-organism single-cell RNA-seq maps from two worm species. The data show that metacells approximate the expression distribution in a surprisingly accurate fashion, dissecting the dataset into truly homogenous local neighborhoods and providing quantitative building blocks for exploring the global expression manifold. We suggest that MetaCell provides, especially as the size of single-cell atlases increases, an attractive universal first layer of analysis on top of which quantitative and dynamic analysis can be developed further.

## Results

### Overview of the MetaCell method

**Fig. 1:**
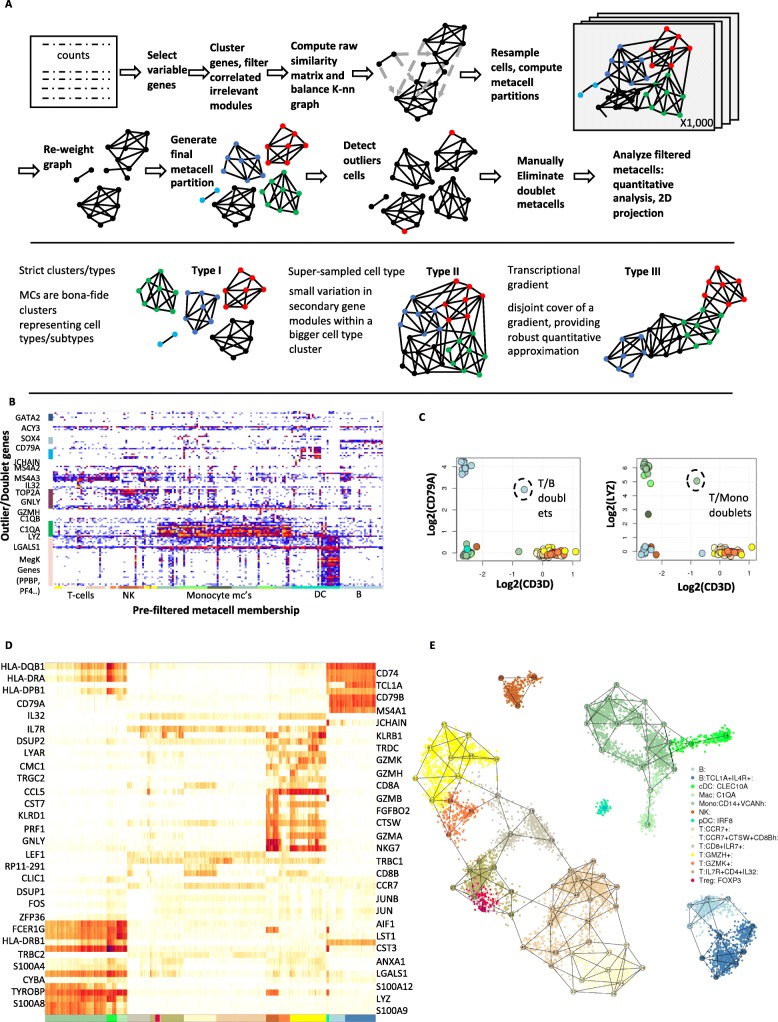
Metacell analysis of the PBMC 8K dataset. **a** Schematics of the MC algorithmic pipeline. **b** Outlier/rare cells matrix showing color-coded number of UMIs per cells (columns) for which at least one gene (rows) was shown to be expressed significantly beyond its MC expected number of UMIs. Outlier/rare cells are ordered according to the annotation of the MC containing them (bottom color-coded bars). **c** Shown are log-fold-enrichment (lfp, methods) values for metacells, color-coded according to initial cell type annotation, comparing the T cell marker (CD3D) to a B cell (CD79A) and myeloid (LYZ) markers. **d** Heat map shows enrichment values for metacells (columns) and their maximally enriched gene markers. **e** Shown is the MC adjacency graph (numbered nodes connected by edges), color-coded according to their cell type and transcriptional state annotation. Cells are shown as small color-coded points localized according to the coordinates of MCs adjacent to them. Additional file 2: Figure S3 shows the adjacency matrix that was used to generate the projection

The MetaCell construction pipeline partitions an scRNA-seq dataset into disjoint cell groups using a non-parametric graph algorithm (Fig. 1a). This partition provides initial metacells that can later be pruned and filtered for homogeneity. First, feature genes are selected and used to compute a raw cell-to-cell similarity matrix *S*. Second, a balanced *K*-nn similarity graph *G* is constructed, connecting pairs of cells that represent reciprocally high-ranking neighbors. In contrast to a *K*-nn graph built directly from *S*, which can be highly non-symmetric, the graph *G* has more balanced ingoing and outgoing degrees. Third, *G* is subsampled multiple times, and each time the graph is partitioned into dense subgraphs using an efficient algorithm. The number of times each pair of cells co-occurred in the same subgraph is used to define the resampled graph *G*^*boot*^. After these three layers of cell-to-cell similarity matrix normalization, the metacell solution is derived using a graph partitioning algorithm applied to *G*^*boot*^.

After the initial construction of a graph partition, we perform pruning and filtering of metacells to increase their homogeneity. We do not enforce a strict multinomial model as empirical data only approximately supports it (see in-depth analysis below), and instead ensure that clear violations of homogeneity are filtered. First, outliers are detected and filtered using a simple parametric test for gene overexpression compared to their metacell. Second, the metacells’ homogeneity is verified, and metacells showing strong sub-cluster structure are split. In practice, splitting is rarely necessary, but outlier detection may require parameter tuning (see Additional file 1: Table S1). Third, metacells representing doublets (composed of groups of profiles that share a similar doublet mixture) are searched for and filtered in a supervised manner. Most of the doublets, however, are identified as such during the outlier filtering stage.

Figure 1a illustrates different types of metacells that are obtained in different experimental scenarios. When a limited number of single cells are sampled from a highly distinct transcriptional behavior, a metacell may define a completely isolated cluster (type I MCs). When a larger number of cells are sampled from a cell state, several metacells may cover it, defining variation in secondary biological behaviors (e.g., cell cycle) or even equivalent transcriptional distributions (type II MCs). More informatively, when sampling a dynamic process that induces a transcriptional gradient across single cells, metacells may create a piecewise approximation of the process (type III MCs). We note that in the latter cases, the MC cover need not be uniquely defined.

Based on a filtered set of metacells, we can robustly explore the scRNA-seq transcription manifold, performing marker-based annotation of the metacells, grouping of metacells into higher-order clusters, and visualizing the data by projecting metacells onto a 2D space. In essence, the analysis downstream the identification of metacells is similar to common scRNA-seq strategies, but replacing sparse single cells, or smoothed single cells, with fewer but more robust metacell profiles.

MetaCell is readily applicable as an R/C++ package and is scalable to large datasets. The full method and implementation details are given in the “Methods” section. Information on feature selection is provided in Additional file 3.

### Metacells eliminate outliers and reconstruct cell type structure in PBMC data

We first illustrate the use of the MetaCell algorithm and pipeline through re-analysis of a small (*n* = 8276) dataset of PBMC scRNA-seq profiles sampled from a healthy donor and downloaded from the 10x website. In a pre-processing step (see Additional file 2: Figure S1), we removed cells with less than 800 UMIs (Additional file 2: Figure S1A) and several non-coding RNAs linked with stress or apoptotic signatures (“blacklisted genes”) (Additional file 2: Figure S1B). We then applied the metacell construction pipeline as outlined above, using 816 high variance genes as features (Additional file 2: Figure S1C, excluding ribosomal proteins) and deriving an initial set of 82 MCs following 1000 resampling iterations using *K* = 100. The MC outlier/rare cell detection screen then identified 182 cells with at least one outlier gene (8-fold or more enrichment over the respective MC model) (Fig. 1b, Additional file 2: Figure S2). Most outlier cells showed potential doublet profiles, co-expressing genes associated with two different cell types. For example, this effect was notable in the association of a coherent megakaryocytic gene module (including PF4, PPBP and more genes) with signatures linked to other cell types. In fact, pure megakaryocyte expression profiles are very rare in the data, and the MC outlier analysis highlights their identification (Additional file 2: Figure S2). In addition to potential doublets, outlier cells also included representatives of rare cell types, including cells expressing progenitor markers (SOX4 [36]) or eosinophilic markers (MS4A2, MS4A3 [37]).

Doublet outlier cells are observed when two cell types are mixed rarely in the data, thereby contaminating a metacell associated with one cell type with a few mixed signatures. More frequent doublet scenarios can give rise to homogeneous doublet MCs, as we observed for two cases combining expression of T cell marker genes (e.g., CD3D) with either B cell (CD79A) or monocyte (LYZ) markers (Fig. 1c). Following the removal of these two doublet MCs, we ended up with a model organizing 7901 cells in 80 MCs (45–176 cells per MC, median size 95 cells) and marking 375 cells as outliers or doublets. This model was annotated using enriched gene markers (Additional file 2: Figure S3) and visualized using a marker heat map (Fig. 1d) and a 2D layout computed from the MC adjacency matrix (Fig. 1e). This visualization organizes transcriptional states in the blood into clear cell type groups representing T, NK, and B cells; monocytes/macrophages; and DC populations. Within these cell types, the maps show additional structure. For example, T cells were organized into CD8+ effector states (marked by GZMH and additional genes), CD8+ pre-effector states (marked by GZMK+), CCR7+ CD8+ cells with variable degree of cathepsin-W (CTSW) expression, naïve CD8+ cells (IL7R+), and CD4+ cells showing some activation of Treg genes (FOXP3+). Overall, when sampling at a depth of 8000 cells, the metacell analysis allowed for robust identification of cell types and initial modeling of gene expression distribution within them. Additional coverage can lead to refined modeling of transcriptional distributions within cell types as we shall demonstrate below, but first, we will use this basic model to evaluate the similarity structure and homogeneity of metacells.

### MetaCell graphs define a symmetrized and modular adjacency structure between MCs

**Fig. 2:**
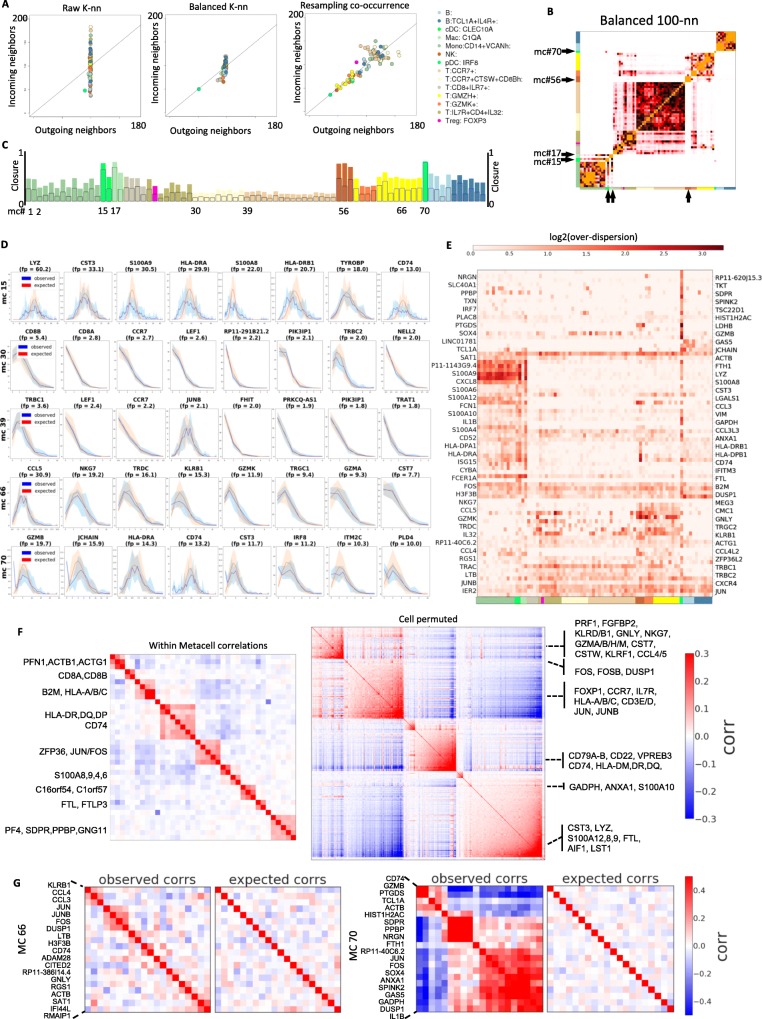
Evaluation of within-MC transcriptional homogeneity. **a** Shown are the number of incoming and outgoing neighbors (or degree) per cell, averaged over metacells that are color-coded by cell type annotation as in Fig. 1. The data represent the raw *K*-nn similarity graph (left), balanced MC graph (center), and resampled co-occurrence graph (right). **b** Heat map summarizing the number of edges in the balanced MC graph that link two cells associated with different MCs. Similar matrices generated based on the raw and co-occurrence graphs are shown in Additional file 2: Figure S4. **c** Bar graph shows the closure per MC (fraction of intra-MC edges out of all edges linking cells in the MC). **d** Observed (blue) vs predicted (red, based on binomial model) distributions of down-sampled UMI count per gene within MCs. For each of the 5 MCs depicted, the plots show binomial fit for the top 8 enriched genes. Intervals give 10th and 90th percentiles over multiple down-samples of the cells within each metacell to uniform total counts. **e** Over-dispersion of genes relative to a binomial model across genes and MCs. Colors encode ratio of observed to expected variance across genes (rows) and MCs (columns). Only genes and MCs manifesting high over-dispersion are shown. **f** Residual within-MC correlation patterns compared with global correlation patterns. Within-MC correlation matrix (left) was computed by averaging gene-gene correlation matrices across MCs, where each matrix was computed using log-transformed UMIs over down-sampled cells. Global correlation matrix (right) was computed in the same manner, but following permutation of the MC assignment labels. For both matrices, only genes manifesting strong correlations are shown. **g** Examples of residual intra-MC correlated genes, showing observed correlations (Pearson on log-transformed down-sampled UMIs) compared to correlations expected by sampling from a multinomial. MC #66 show weak residual correlations reflecting mostly stress genes. MC #70 shows stronger residual correlations, reflecting residual intra-MC variation

The impact of the procedures transforming raw cell-to-cell similarities to the MetaCell graph are illustrated for the PBMC data in Fig. 2a. The initial distribution of in-degree in the *K*-nn graph (*Y* axis, left panel) shows significant variation, which is corrected by a graph balancing procedure (middle panel). The resampled co-occurrence graph maintains the linkage between in and out degrees, but decreases the connectivity of the graph for specific cell types that are under-sampled (right panel). This actual effect of these transformations on cell type modularity is analyzed through the MC adjacency matrices that summarize connectivity between cells within each pair of MCs. Comparing raw *K*-nn, balanced, and resampled MC similarities (Fig. 2b and compare Additional file 2: Figure S4) shows for example initial spurious connectivity from NK cells (MC #56) toward T cells and from pDCs (MC #70) toward multiple cell types in the raw matrix, which are eliminated in the balanced and resampled matrices. This comparison also highlights cases of myeloid MCs connecting a large group of monocyte MCs and cDCs (#15) or monocytes and macrophages (#17), that provide better separation with the more differentiated MCs in the balanced and resampled matrices. The resampled matrix in particular provides improved modularity within the large group of T cell MCs, for example, grouping of CCR7+ T cell MCs into distinctive clusters. In summary, in a typical scRNA-seq dataset, the combination of abundant and rare states leads to an asymmetric *K*-nn structure linking rare cells with hubs within large clusters, and the MetaCell graph balancing procedure alleviates such effects. The approach is somewhat similar to methods using mutual *K*-nn analysis to normalize batch effects [38, 39], or more generally to approaches using symmetrization of the *K*-nn graph to facilitate dimensionality reduction [40].

### Comparing metacells’ graph closure with their transcriptional homogeneity

To quantify the accuracy of the MC approximation to the similarity graph, we computed the fraction of *K*-nn similarities captured within each MC, which we refer to here as the MC’s *closure*. As shown in Fig. 2c, the level of closure varies considerably between cell types. Distinct and low abundance cell types (type I MCs) can show very high closure (up to 100%), while multiple MCs that cover abundant cell types (type II or III MCs) show overall low closure (as low as 10% within-MC adjacencies, 20–30% within the three most linked MCs). Imperfect closure may suggest that the MC partition is suboptimal or, alternatively, that the *K*-nn local similarity structure in large and diffused cell types is covered by multiple, non-maximal but still homogeneous MCs (Type II MCs in Fig. 1a). To test this, we compared the intra-MC UMI distribution to the distribution predicted by a simple multinomial model for specific genes and MCs (Fig. 2d). We found that low closure MCs show high degree of consistency with the multinomial model, confirming their homogeneity. Interestingly, MCs with very high closure may show a reciprocal behavior, where additional high variance is present within *K*-nn consistent clusters (e.g., MC #70; note the bimodal distributions observed for most genes). This analysis highlights a key property of the MC partition: MCs are not maximal, and multiple highly similar MCs which are only weakly separated in the similarity graph can together approximate a larger cluster.

### Multinomial sampling explains most of the intra-MC UMI variance

Systematic screening for genes showing intra-MC over-dispersion (Fig. 2e) provides a global view on the consistency of the PBMC MC cover with simple multinomial sampling. In this screening, MCs containing residual, non-homogeneous structure will be associated with many over-dispersed genes. For example, this analysis associates the dendritic cells MC #70 with over-dispersion of multiple megakaryocyte-associated and other genes. This suggests that these poorly sampled cell types show additional hidden structure and potential remaining outlier cells. The screening also reveals specific genes that are consistently over-dispersed across many MCs, such as the early-immediate response gene module (including the transcription factors JUN, JUNB, FOS). This over-dispersion is consistent with variable levels of activity of this pathway in multiple cell types, perhaps representing technical experimental stress. Other genes are over-dispersed in a cell-type specific fashion, for example cytotoxic (GNLY, CCL5) genes in NK and T subtypes, and MHC-II and LYZ in myeloid cell types. These highly expressed genes may be incompatible with a simple multinomial sampling model, and their analysis may necessitate assuming prior biological variance to allow for over-dispersion. Beyond these specific examples, however, intra-MC distributions for the entire gene set (including genes that were not used as features for defining similarities) are generally well approximated by Poisson sampling with no zero inflation (Additional file 2: Figure S5). Together, the data shows that the degree of residual, intra-MC over-dispersion is relatively low in the PBMC MC cover, so that the variance of most genes is accounted for by a model assuming partition of cells into MCs from which UMIs are multinomially sampled.

Analysis of intra- and inter-MC gene-gene covariance (Fig. 2f) provided an additional avenue for diagnosing structure within and between MCs. We observed persistent intra-MC correlations between a limited set of genes, including the over-dispersed modules of early-immediate genes, MHC class II genes, and S100 genes as well as a correlated gene set including actin-related genes (ACTB, ACTG1, COTL1, PFN1). We did not observe strong intra-MC correlations of cytotoxic and many other functional genes. The scarcity of strong intra-MC gene-gene correlations (see for example Fig. 2g, MC #66) suggests that little residual structure remains within the MCs, and that the dataset is well summarized by the MC profiles. In the few cases where intra-MC correlations are observed (Fig. 2g, MC #70), they indicate the need for a more flexible intra-MC modeling, or alternatively call for deepening the dataset with more cells defining the transcriptional states underlying the MC.

### Metacells are accurate local approximations of the expression manifold

**Fig. 3:**
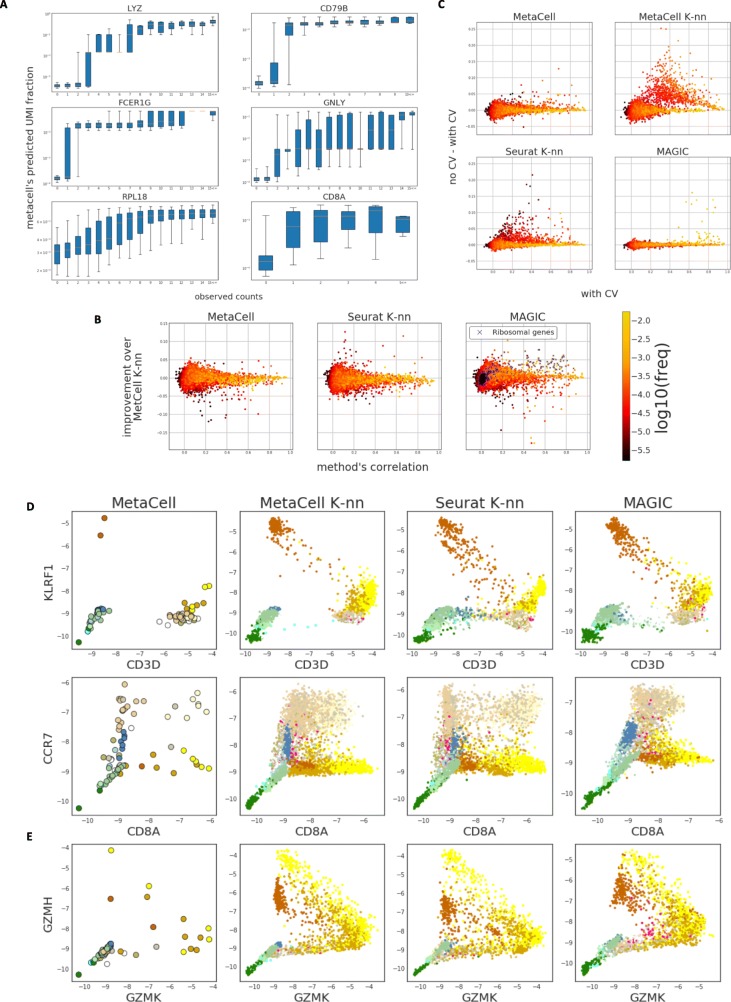
MCs robustly approximate the expression manifold. **a** Boxplots show the distribution of predicted (using MC pool frequencies) UMI fraction per cell stratified according to observed number of UMIs in down-sampled single cells. **b** Shown are per-gene Pearson correlations between predicted and observed gene frequencies for genes, color coded according to the gene’s frequency across all cells. In all cases, predictions are generated using a 100-fold cross-validation scheme (see the “Methods” section for exact description of the procedure and the strategies compared). Predictions using *K*-nns over raw MC similarities (a different neighborhood per cell consisting of its *k* most similar neighbors) are used as reference. It is compared to strategies defining cell neighborhoods using MCs (fixed disjoint grouping of cells), *K*-nn over Seurat distances, and MAGIC distances (weighted neighborhood according to diffusion distances). **c** Similar to panels in **b** but comparing accuracy with and without applying cross validation. Points with high value along the *y* axis represent potential over-fitting. **d**, **e** Per-MC (left most column) or smoothed per-cell (all other columns) expression values for pairs of genes, portraying putative transcriptional gradients

All approaches for analysis of scRNA attempt to describe aspects of the expression manifold, each relying on different assumptions. MetaCell generates a high-resolution partition of the data, thereby focusing on approximating it locally. We tested the quality of this approximation using a cross-validation scheme, in which we predict the expression of each gene using a MetaCell model trained on data from which the gene was left out. Figure 3a illustrates the outcome of such prediction, showing accurate prediction for highly expressed genes and lower accuracy for low-UMI counts, for which sampling variance is high. We wanted to compare these predictions to those obtained using the models that underlie commonly used approaches for scRNA-seq analysis. To this end, we computed the cell-to-cell similarity matrices inferred by Seurat’s [12] PCA-based approach and by a diffusion strategy as implemented in MAGIC [25]. We also included in the comparison the similarity matrix *S* initiating the MetaCell balancing process. For all similarities, we employed the same cross-validation scheme that was applied to the MetaCell model, and computed local predictions by averaging 50 nearest neighbors for Seurat and *S*, and weighting all cells by their similarities for MAGIC (see the “Methods” section for a complete description).

Differences in prediction accuracy should reflect the different similarity measures employed by each method as well as the effect of disjoint partitioning applied in MetaCell. In theory, the partitioning strategy should provide less modeling flexibility compared to approaches that compute cell-specific neighborhoods. The latter effect should be particularly noticeable when several MCs discretize a continuum, such as differentiation trajectory (type III MCs, Fig. 1a). In practice, we observed relatively mild differences between the different approximations (Fig. 3b), with very few genes losing accuracy when MCs are used. Moreover, analysis of the gain in accuracy when including all genes in the models (Fig. 3c) suggested that MetaCell is significantly less exposed to over-fitting than the *K*-nn approaches. The diffusion-based smoothing approach showed minimal overfitting, but also loss of accuracy (Fig. 3c). Overall, the nearly multinomial intra-MC UMI distribution observed above and the minimal loss of predictive power entailed by the MetaCell disjoint partition, together suggest that MCs succeed in capturing most of the biological variation in the data, while eliminating most of the sampling noise.

### Metacells avoid artefactual gradient effects

We showed that the cell partitioning induced by MetaCell does not decrease local approximation accuracy and that, in fact, it even reduces the model’s tendency to over-fit the data. We speculated that another advantage of partitioning would be robustness to over-smoothing. The discussion about over-smoothing recently arose in the context of evaluating scRNA-seq imputation methods, i.e., methods that use the covariance patterns measured across multiple cells and genes to refine per-gene, per-cell measurements (reviewed here [41]). Most imputation methods are local in the sense that they impute gene expression for a cell using its inferred neighborhood. It has been observed [27, 28] that in some cases imputation tends to enforce spurious proximities between cells, which in turn manifest as artefactual gradients, i.e., discrete states pertaining to be a series of cells gradually modulating expression of certain genes along a temporal process or a spatial axis. While over-smoothing is detected directly when evaluating imputation methods, it is in fact a potential concern with any model regardless of its downstream application, and stems from the manner in which cell-cell similarities are defined.

We evaluated the susceptibility of the MetaCell model to over-smoothing using the expression predictions obtained in the previous section (the version without cross-validation), comparing the different similarity structures included in that experiment. Our results support the robustness of MetaCell to artefactual gradients (Fig. 3d). For example, NK cells are known to be characterized by high levels of KLRF1, but do not express the T cell classical marker CD3 (Fig. 3d, top). Smoothing based on *K*-nn similarity structures (MetaCell’s *K*-nn or Seurat’s) or on diffusion similarities (MAGIC’s) gives rise to phantom gradients that can be interpreted erroneously, for example, as supporting differentiation of NK to T cells or vice versa. The MC statistics generate a much less detailed, but likely more realistic map of joint CD3D/KLRF1 expression. Similar phantom gradients are observed when analyzing CCR7+ CD8+ and CCR7+ CD8− cells (Fig. 3d, bottom). On the other hand, the MC model does reveal expression gradients in cases where sampling adequately supports them, such as in the trade-off expression of GZMK+ and GZMH+ in T cells (Fig. 3e). These quantitative gradients are refined in the denser dataset we analyze below. Robust modeling of transcriptional gradients by MCs is also demonstrated on simulated data (Additional file 2: Figure S6).

### Dissecting complex cell type hierarchies with MetaCell

**Fig. 4:**
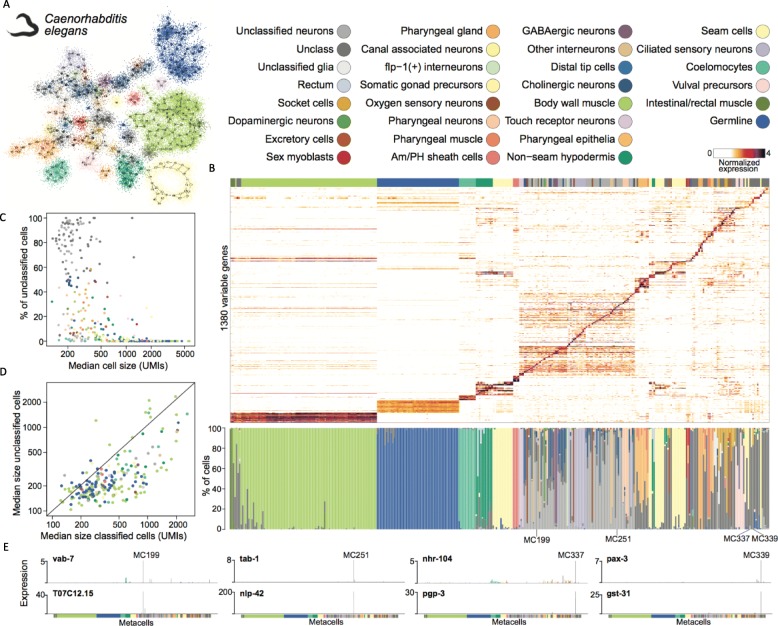
MC analysis of a whole-organism single-cell dataset. **a** 2D projection of *C. elegans* metacells and single cells, color-coded according to the most frequent cell type based on the classification from Cao et al. **b** Top—normalized expression of 1380 highly variable genes across 38,159 *C. elegans* single cells (columns), sorted by metacell. Bottom—bar plot showing for each metacell the single-cell composition of the different originally classified cell types. **c** Relationship between the metacell median cell size (UMIs/cell) and the fraction of cells originally labeled as “unclassified” in Cao et al. **d** Comparison of the median sizes (UMIs/cell) of originally unclassified cells versus classified cells in each metacell. **e** Expression (molecules/10,000 UMIs) of selected marker transcription factors (top row) and effector genes (bottom row) across all metacells, supporting high transcriptional specificity for four examples of metacells containing a high fraction (> 80%) of originally unclassified cells

We tested the scaling of MetaCell to datasets consisting of a large number of cell types and high variability in the total number of UMIs per single cell. To this end, we revisited two whole-organism scRNA-seq studies dissecting *C. elegans* (*Caenorhabditis elegans*) [42] and Planaria (*Schmidtea mediterranea*) [43]. For *C. elegans*, we compared the derived MC partition (349 MCs) (Fig. 4a, Additional file 2: Figure S7) to the published model grouping cells into 27 major cell types (Fig. 4b). We observed a high degree of consistency between the two models in classifying the major cell types, with higher resolution in dissecting cell types into subtypes using MCs (e.g., for body wall muscles, seam cells and more). Importantly, we observed a large number of cells labeled originally as “unclassified” or “unclassified neurons/glia” that were organized within coherent MCs. Some of these MCs were dominated completely or almost completely by unclassified cells. Moreover, we observed a negative correlation between the median number of UMIs per cell in a metacell and the fraction of unclassified cells within it (Fig. 4c). Comparing the number of UMIs per cell within MCs showed consistently lower UMI counts for unclassified cells (Fig. 4d). The transcriptional specificity of MCs containing large fractions of unclassified cells was uniformly high, as confirmed by observation of co-expression of specific transcription factors and genes within such MCs (Fig. 4e). Similarly, MetaCell analysis of the rich whole-organism cell type map of Planaria showed extensive consistency between the MC partition (564 MCs) and the iterative and highly supervised clustering analysis (512 clusters) used to annotate the original map (Additional file 2: Figure S8). In summary, while MetaCell is not designed to perform clustering in its classical sense, a metacell partition facilitates robust and sensitive cell type mapping of scRNA-seq data, in particular when gene expression and cell type sizes are extremely heterogeneous.

### High-resolution analysis of inter- and intra-cell type states in the blood

**Fig. 5:**
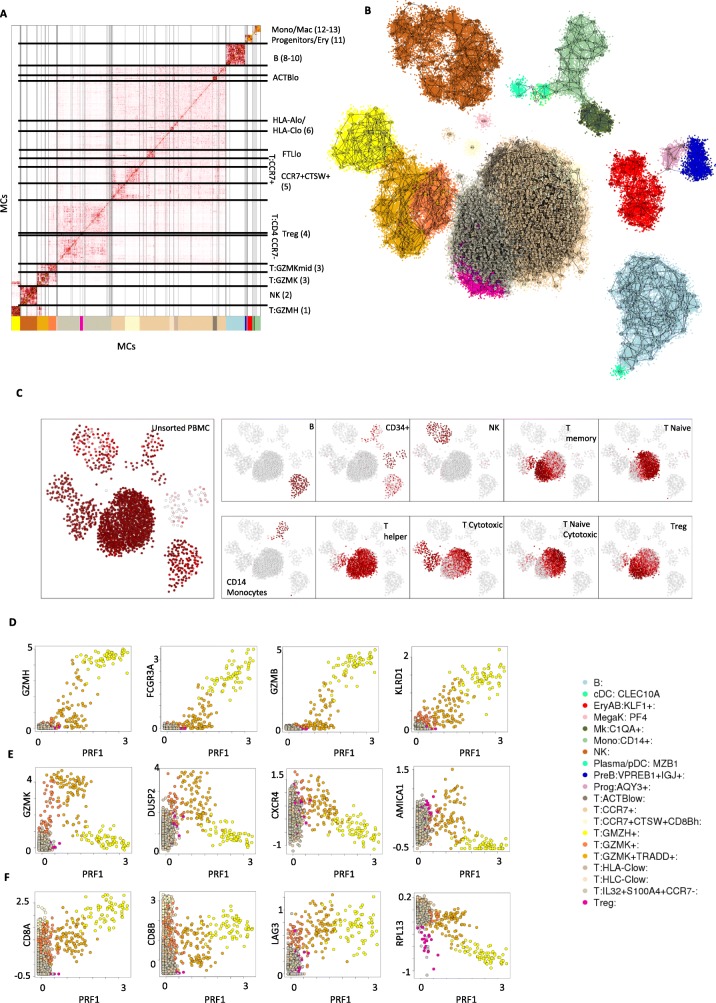
MC analysis of a 160K PBMC multi-batch dataset. **a**, **b** Matrix (**a**) and graph (**b**) visualization for the similarity structure associating MCs in a model characterizing 162,000 PBMCs. Clusters in the MC matrix are used for linking specific groups of MCs with specific annotation and for color coding. **c** Shown are the fraction of cells from different sorting batches per MC, color coded white to red to black and visualized using the MC 2D projection as shown in Fig. 4B. **d** Shown are lfp values for MCs in the PBMC 160K model, comparing intensity of Perforin expression (*X* axis) to several genes correlated with the CD8+ effector program. **e** Similar to **d** for genes showing transient activation during the effector program build-up. **f** Similar to **d** for CD8 genes, LAG3 (a T cell exhaustion marker) and a representative ribosomal protein gene

We next tested the scaling of the MetaCell algorithmic pipeline when applied to datasets sampling deeply a relatively small number of cell types by analyzing RNA from 160K single blood cells, including 68K unsorted PMBCs and 94K cells from ten different bead-enriched populations [44]. We hypothesized that, with increased number of cells, we could derive MCs with enhanced quantitative resolution and increased homogeneity, thereby allowing a more accurate identification of regulatory states and differentiation gradients in the blood. We derived a model organizing 157,701 cells in 1906 metacells, identifying 4475 cells as outliers. Figure 5a summarizes the similarity structure over the inferred MCs, indicating partitioning of the dataset into T cells, NK cells, B cells, myeloid cells, megakaryocytes, and progenitor cells. In-depth analysis of the emerging cluster and sub-cluster structure in this matrix allowed us to identify groups of related MCs for further analysis, in many cases providing us with the ability to zoom into transcriptional programs (cell groups numbered 1–13 on Fig. 5a) within large-scale clusters that were identified in the global metacell 2D projection graph (Fig. 5b). Visualization of genes that were specifically enriched in such programs demonstrates both bimodal markers and putative quantitative gradients organizing MCs within and between types (Additional file 2: Figure S9). For example, we observed the correlated (and bifurcated) intensity of CD8A and CD8B expression in cytotoxic and memory T cells, the variable MHC-I expression (HLA-A,HLA-C) in different cell sub-types (group [6]), variable levels of granzyme K and granzyme H expression along a putative cytotoxic gradient of CD8+ cells (groups [1], [3]), and a group of MCs expressing cathepsin W and CCR7+ but without the cytotoxic gene module (group [5]). The analysis of specific gene families (see Additional file 2: Figure S10) illustrates how multiple effector genes are activated in different cell types in a convergent fashion (Additional file 2: Figure S10A). Analysis of transcription factor expression across the different subtypes (Additional file 2: Figure S10B) provided an initial blueprint for the regulatory mechanisms defining the observed transcriptional states. Importantly, the integration of different sorting batches allowed for enhanced resolution in several hematopoietic lineages, in particular CD34+ progenitor cells (Fig. 5a, group [11]). Nevertheless, all MCs within the non-progenitor cell types represented a balanced mixture of sorted and non-sorted batches (Fig. 5c). We note that the metacells produced by MetaCell’s specialized partition algorithm cannot be reproduced by conventional clustering, at least when used naively. We demonstrate this by clustering the PBMCs with Seurat using parameters that force fine clustering, generating 817 clusters (Additional file 2: Figure S11). As shown in Additional file 2: Figure S11A, the MC partition is consistent with these fine clusters at the level of the coarse-grained cell types, but not at higher resolutions. The fine clustering solution generates clusters that are likely to be overfitting specific genes (Additional file 2: Figure S11B). In summary, for the densely covered, multi-batch 160,000 PBMC datasets, MetaCell provides analysts with a platform for distinguishing cell types and their internal hierarchies, and a robust scheme for characterizing quantitative expression gradients with guarantees against spurious smoothing effects.

### Using MCs to define gradients of CD8+ effector T cell activation

Finally, we demonstrate the potential of applying MetaCell for in-depth analysis of differentiation gradients through analysis of the transcriptional signatures in effector CD8+ T cells. Activation of the T cell effector program ultimately depends on expression of units of the cytotoxic granule (granzymes, cathepsins, granulysin) and of the machinery required for perforating target cells (e.g., perforin) [45]. Elevated expression of Perforin 1 (PRF1) is indeed observed in a subset of the CD8+ MCs, spanning a spectrum of intensity from background level to 10-fold enrichment over it. We observed PRF1 enrichment to correlate strongly with multiple additional effector genes, for example granzyme H and B, FCGR3A, and KLRD1 (Fig. 5d), consistent with the idea of a spectrum of transcriptional states with variable effector gene toolkit expression in the blood. Remarkably, we identified a second set of genes showing elevated expression in MCs with low-to-intermediate effector program expression (Fig. 5e), including most notably granzyme K (GZMK) and the phosphatase DUSP2, but possibly also the chemokine receptor CXCR4 and the adhesion/motility molecule AMICA1/JAML. The effector program expression gradient was also associated with decrease in relative housekeeping gene expression (e.g., ribosomal proteins, Fig. 5f). We note that the association between the transcriptional gradient of effector genes and temporal or differentiation processes cannot be assumed immediately. It is nevertheless tempting to suggest that effector program activation involves transient expression of the GZMK-linked genes observed here, suggesting several experimental directions for follow-up toward a better understanding of T cell commitment and regulation in the blood and other organs, and in particular within tumors [29, 46].

## Discussion and conclusions

We introduce here the use of metacells for analyzing scRNA-seq data. Metacells are defined as groups of single-cell profiles that ideally represent re-sampling from the same cellular state. In practice, we compute MCs as a graph partition using adequately processed similarities between single-cell profiles. We demonstrate that in real data, we can construct partitions such that the intra-MC UMI distribution can be approximated as sparse multinomial sample, representing sampling from a highly specific transcriptional state with no significant additional variance. We show how to screen for MCs with over-dispersion or residual pairwise gene correlations, reflecting deviation from this model and residual intra-MC biological variation. We then demonstrate how the MCs can be used for in-depth exploration of large data sets involving either a rich set of cell types (whole organism) or a limited and over-sampled set (PBMCs). The analysis methodology we advocate involves direct inspection of the MC adjacency matrix, which provides analysts with complete information about cell type hierarchy and supports clustering at appropriate resolution. Combined with visual examination of correlation patterns between MC-enriched genes, the result is a detailed and unbiased characterization of cell types and expression gradients that we have already used in several challenging analysis scenarios [31–35].

The main property that makes metacells a powerful analysis tool is their ability to increase the signal-to-noise ratio in the data without introducing biases stemming from mistaken modeling assumptions or over-smoothing of the data. The only manipulation performed by MetaCell on the data is the pooling of highly similar cells, thereby forming a partition of the data. The analyses we present show that, despite enforcing this partitioning, a metacell cover provides accurate local approximations of the expression manifold. At the same time, partitioning entails multiple advantages. Statistically, it greatly reduces the effective number of parameters of the model, making it less prone to over-fitting and to over-smoothing compared with naïve smoothing approaches. For the analyst, it allows for the characterization of well-defined, discrete and highly granular states in a conservative and easy-to-interpret framework.

In cases where residual intra-MC structure is detected in the cover, additional cells can be sampled to refine the MC cover and tighten the approximation. Fundamentally however, in any realistic data set, there will always remain some under-sampled behaviors regardless of sampling depth, and our current model will not provide a constructive approach for understanding such behaviors beyond signaling them out as non-homogeneous. Fitting more flexible intra-MC models, capable of accounting for not only sampling noise but also convergent processes such as cell cycle or stress [47, 48], or embedding the metacells in hierarchical or multi-resolution structures [49, 50] should allow for more efficient extraction of the signals of interest. We view the integration of such models as an important future extension of this work.

## Methods

### Notation and definitions

We assume raw scRNA-seq reads are mapped to genome sequences and assigned to cell barcodes and unique molecular identifiers (UMI) using pipelines that eliminate most of the UMI duplications induced by PCR and sequencing errors. We summarize all UMIs in the molecule count matrix *U* = [*u*_*gi*_] on genes *g* ∈ *G* and cells *i* ∈ *I*. We define *u*_*g*_ as the total molecule count for gene g on the raw count matrix, and *u*_*i*_ as the total number of molecules for a cell (sometime referred to as the cell’s *depth*). The procedures below are designed to robustly define a metacell partition over the cells, which is denoted by a set of cell subsets *M*_*k*_ and a set of outliers *O* such that $\left(\bigcup \limits_k{M}_k\right)\cup O=I$.

We assume a set of gene features F ⊆ G is specified and focus our analysis on a similarity graph between cells derived using data from these features (see below). We discuss several strategies for selecting genes in Additional file 3. We note that our features represent individual genes rather than principle components or other forms of reduced dimensions. This enables some direct approaches to testing and correcting the gene expression distributions within metacells. It also forces the modeling of similarities and derivation of metacells to work over high-dimensional spaces and to account for noise and sparse data directly. Applying the metacell algorithmic pipeline to similarity structures derived using popular dimensionality reduction techniques is easily applicable as well, as we demonstrate in the results section.

### The metacell balanced *K*-nn cell similarity graph

A well-founded parametric generative model for scRNA-seq data is currently missing, mainly due to the limited understanding of the biological variation in transcriptional states within different cell populations, and the remarkable diversity of coupled (e.g., developmental) and uncoupled (e.g., cell cycle, stress) biological processes that are captured in typical single-cell RNA-seq maps. We therefore use a simple non-parametric approach for modeling raw pairwise local similarities, which is then refined by additional analysis of the derived cell *K*-nn similarity structure. We transform the raw UMI count *U* on the gene features *F* as *U* ′  = [*u*′_*gi*_] = [*log*_2_(*ϵ* + *u*_*gi*_)]_*g* ∈ *F*_ and compute the raw similarity matrix using the Pearson correlations on the transformed features *R* = [*r*(*u*′_*gi*_, *u*′_*gj*_)]_*ij*_. A simple variation on this procedure may include prior normalization of the *U* matrix by down-sampling (sampling min(*u*_*i*_) UMIs from each cell without replacement) so as to avoid biases associated with improved accuracy (and thereby higher similarity) between deeper UMI profiles. We however avoid down-sampling when the distribution of the number of UMIs per cell is highly variable and correct for the sampling bias when manipulating the similarity graph as described below.

Next, we use the raw similarity matrix *R* to generate a weighted adjacency matrix for a directed cell graph, in which a heavy edge from cell *i* to cell *j* indicates strong attraction of the former to the latter. We first perform a non-parametric transformation by computing *S* = [*s*_*ij*_] = [*rank*_*j*_(*r*_*ij*_)]. Here *rank* is the ranking function, and each row represents the order of similarity between all cells *j* and a specific cell *i*. The *S* matrix is highly non-symmetric, for example when the similarities going from an outlier cell are linking it to members of a large, homogeneous, and highly connected cell group. To better control for such effects, we perform the following *balancing* operation. We first symmetrize *S* by multiplying ranks *s*_*ij*_ ∗ *s*_*ji*_, followed by initial regularization of edges using a threshold *αK*^2^ (setting *α* = 10 by default) on the rank product:

$$ \left[{s}_{ij}^1\right]=\left[\mathit{\max}\left(\alpha {K}^2-{s}_{ij}\ast {s}_{ji},0\right)\right] $$

We then perform two rounds of additional regularization, first keeping maximum scoring *βK* incoming edges for each node (*β* = 3 by default):

$$ \left[{s}_{ij}^2\right]=\left[\mathit{\max}\left(\beta K-\mathit{\operatorname{ran}}{k}_i\left({s}_{ij}^1\right),0\right)\right] $$

and then further filtering to keep maximum K outgoing edges for each node:

$$ \left[{a}_{ij}\right]=\left[\mathit{\max}\left(K-\mathit{\operatorname{ran}}{k}_j\left({s}_{ij}^2\right),0\right)\right] $$

A weighted directed graph G is then constructed using [*a*_*ij*_] as the weighted adjacency matrix. Note that nodes with degrees lower than *K* are possible following this procedure, since outlier cells may become disconnected or poorly connected during the balancing operations.

### Seeding and optimizing graph partitions

We partition the balanced similarity graph G into dense subgraphs using an adaptation of *k*-means to graphs. Let the parameter *K* define the typical desired size of subgraphs in the partition (which is also the maximum outdegree of the graph *G* as constructed). Denote by *N*^out^(*i*) the set of graphic outgoing neighbors of *i*. We initialize an empty assignment of cells to subgraphs *mc*(*i*) =  − 1, define the set of covered nodes as *C* = {*i* | *mc*(*i*) >  − 1} and the *cover-free* score for each node as *f*(*i*) = |*N*^out^(*i*) – *C*|. We then sample subgraph seeds using an iterative procedure:

- Initialize *k =* 0
- While $$ \underset{i}{\max }\ f(i)> size\_\mathit{\min} $$ do:
  - sample a new seed cell *j* by drawing a sample from cells in *I* − *C* with weights proportional to *f*(*i*)^3^
  - update *mc*(*u*) = *k for u* = *j*, *u* ∈ *N*^*out*^(*j*) − *C*
  - Increment *k* and update *C*, *f*.

We terminate seeding using a minimum subgraph size parameter *size* _  *min*  < *K*. When we meet the stop criterion, cells that are not associated with a seed (i.e., cells for which *mc*(*i*) =  − 1) have at most *size* _ *min* uncovered neighbors and in particular will almost always have at least one covered neighbor (since the degree in the balanced graph is typically *K*).

The seeding step produces an initial set of subgraphs *M*_*k*_ = {*i* | *mc*(*i*) = *k*} that forms a basis for further optimization. Define the outgoing association of each cell to a subgraph as $w{o}_{ik}={\sum}_{\left\{j\in {N}^{out}(i)\cap {M}_k\right\}}{a}_{ij}$ (recall *a* are the graph weights), and analogously the incoming subgraph association for each cell as $w{i}_{ik}={\sum}_{\left\{j\in {N}^{in}(i)\cap {M}_k\right\}}{a}_{ji}$. The combined cell-to-subgraph association is computed by multiplying the outgoing and incoming weights and normalizing by the respective subgraph size: *w*_*ik*_ = *wi*_*ik*_ *wo*_*ik*_/|*M*_*k*_|^2^. We use this scoring scheme to iteratively optimize the initial graph cover, and ensure that it includes all cells:

- Until convergence:
  - Select a cell *i*
  - Reassign *mc*(*i*) = *argmax*_*k*_ *w*_*ik*_
  - Update weights

Convergence is defined by deriving a partition in which all cells are associated with their highest scoring subgraph. To enforce convergence (which is not guaranteed to occur in general), we slowly increase the score association between cells and their current subgraph after each reassignment. This is especially useful when a large subset of cells (i.e., larger than *K*) are very homogeneous, which may result in unstable exchange of nodes between several modules covering this subset.

After convergence, there are no formal guarantees on size distribution of the subgraphs produced by the algorithm. Empirically, however, the connectivity of the graph (maximum *K* outgoing edges) and the seeding process promote a relatively uniform cover partition and prevent convergence toward solutions with very large subgraphs. Rare cases of cells that reside in connected components whose size is smaller than *size* _ *min* and were left uncovered during seeding are defined as outliers.

Importantly, the complexity of the entire procedure (seeding and optimization) is linear in the number of cells and the maximum degree *K* (or alternatively, linear in the number of edges in the graph). An efficient implementation of the algorithm therefore scales well to large datasets, as does its integration within an extensive resampling strategy, as we discuss next.

### Resampling graph partitions and computing metacells

We improve the robustness of the above randomized graph partition algorithm using a resampling approach. Given the balanced graph *G*, we generate a series of subgraphs *b* = 1. . *N*_*B*_ (typically *N*_*B*_ = 500) by sampling cells independently without replacement with probability *ρ* (typically *ρ* = 0.75) and adding all edges connecting them, forming *G*^*b*^ = (*V*^*b*^, *E*^*b*^), *V*^*b*^ ⊂ *V*, *E*^*b*^ ⊂ *E*. For each resampled *G*^*b*^, we apply the partition algorithm, thereby generating a set of partial graph partitions *mc*^*b*^(*i*) *for each i* ∈ *V*^*b*^. We summarize all partitions using the matrices *O* = [*o*_*ij*_] and *C* = [*c*_*ij*_], specifying how many times the pair of cells *i*, *j* were resampled together, and how many times they were both assigned to the same subgraph in the resampled partition, respectively. We then define the resampled co-occurrence matrix as ${S}^{boot}=\left[{s}_{ij}^{boot}\right]=\left[{c}_{ij}/{o}_{ij}\right]$.

The values in *S*^*boot*^ are now used to compute a weighted, non-directed graph, discarding the original correlation distances. We compute for each cell *i* the value of the *K*^*core*^ (typically 30) highest frequency neighbors (denoted *T*_*i*_) and then define a co-occurrence threshold for each pair of cells using the maximal of the two critical values multiplied by a factor *T*_*ij*_ =  *max* (*T*_*i*_, *T*_*j*_) ∗ 0.5. Pairs with ${S}_{ij}^{boot}>{T}_{ij}$ are used as the edges in a new graph denoted as *G*^*boot*^ on all cells. Note that *G*^*boot*^ is still of non homogeneous degrees, as setting fixed thresholds on edges implies that nodes in large and diffused clusters will have a lower *T*_*i*_ values and thereby higher degree than nodes in tight and robust clusters that always cluster in the same subgraphs. The parameter *K*^*core*^ provides users of the algorithm with flexible control over the degrees in the derived graph. The final partition solution is obtained by re-applying the same partition algorithm on the graph *G*^*boot*^, resulting in a new set of subgraphs *M*_*i*_ and a potential list of outliers. This solution is subject to further filtering and verification, as described next.

### Filtering clear parametric outliers from a metacell cover

As commented above, even though we lack a proper parametric model for single-cell RNA-seq, our idealized metacell cover is expected to group together single-cell profiles that are approximately consistent with multinomial sampling. Testing a given metacell cover for gross inconsistencies with this assumption can help detecting outlier cells emerging from experimental errors (such as doublets), as well as diagnose rare states that are not sufficiently abundant to define a separate metacell. We currently approach this detection problem heuristically, by summarizing the metacell’s pool frequencies:

$$ {u}_k=\sum \limits_{i\in {M}_k}{u}_i $$

$$ {p}_{gk}=\frac{1}{u_k}{\sum}_{\left\{i\in {M}_k\right\}}{u}_{gi} $$

and computing an approximate, regularized observed/expected value for each gene and cell:

$$ {f}_{gi}={\log}_2\left(\frac{1+{u}_{gi}}{1+{u}_i{p}_{gk}}\right),i\in {M}_k $$

Note that the regularization (adding 1 to observed and expected count) implies that high fold change values (e.g., > 2) cannot be attained for genes with very low overall UMI counts. However, this regularization is sufficient to ensure robust detection of clear outliers. Cells with one or more genes showing high *f*_*gi*_ values are labeled as potential outliers and removed from their metacell cover prior to in-depth quantitative analysis of the model.

### Verifying metacells homogeneity

Outlier filtering does not guarantee metacell homogeneity in cases where two distinct and significantly separated transcriptional states are grouped together. To screen for such scenarios, we attempt to cluster cells within each metacell *M*_*k*_ de novo. Clustering is performed by applying the DBSCAN density-based clustering algorithm to the intra-metacell similarity matrix, computed as the correlation distances described above but restricted to genes exhibiting mildly high intra-metacell variance (normalized variance/mean > 1.2). If more than one cluster is detected, we split the metacell accordingly. In practice, metacells almost never include hidden sub-clusters and testing for splits is used mostly for validation purposes.

### Defining the metacell gene expression profile

We approximate the gene expression intensity within each metacell by a regularized geometric mean:

$$ {p}_{gk}=\mathit{\exp}\left[\left(\frac{1}{\left|{M}_k\right|}{\sum}_{\left\{i\in {M}_k\right\}}\log \left(1+{u}_{gi}\right)\right)-1\right]/\left(\frac{1}{\left|{M}_k\right|}{\sum}_{\left\{i\in {M}_k\right\}}{u}_i\right) $$

We then quantify relative expression as the log fold enrichment over the median metacell value:

$$ {\mathrm{lfp}}_{gk}=\mathrm{lo}{\mathrm{g}}_2\left(\left({p}_{gk}+\epsilon \right)/\mathrm{media}{\mathrm{n}}_{k\prime}\left({p}_{gk\prime }+\epsilon \right)\right) $$

Note that the lfp values are affected by the composition of metacells in the dataset up to a constant and that *ϵ* (typically set to 10^−4^) should be adapted to the typical total molecule count within a metacell.

### Metacell regularized force directed 2D projection

We use the MetaCell cover to regularize the similarity graph among single cells and therefore simplify their 2D projection as follows. We start by projecting edges in the graph G over metacells:

$$ B=\left[{b}_{ml}\right]=\frac{K^2}{\left|{M}_m|\ast |{M}_l\right|}\sum \limits_{\left\{i\in {M}_m,j\in {M}_l\right\}}\left\lceil {a}_{ij}/C\right\rceil $$

(here *C* = median_*k*_(| *M*_*k*_| ) is a scaling constant). We symmetrize B by replacing it with B′, the sum of its row and column-normalized forms, and retain as candidate edges only pairs for which *b*′_*ml*_ > *T*_*edge*_. We then construct a graph over the metacells *G*^*M*^ = (*M*, *E*^*M*^), by adding the *D* highest scoring candidate edges (if they exist) for each metacell. This results in a graph with maximum degree *D* and any number of connected components. We compute coordinates (*xm*_*k*_, *ym*_*k*_) for each metacell by applying a standard force-directed layout algorithm to the graph *G*^*M*^. We then position cells by averaging the metacell coordinates of their neighbor cells in the original balanced graph *G*, but filter neighbors that define a metacell pair that is not connected in the graph *G*^*M*^. Averaging allows for layout flexibility along one or few edges in the metacell graph when positioning large cell clusters that are dissected by several metacells.

### Implementation

We implemented MetaCell using a combination of C++ and R code. We used parallelization over multi-core machines. On a strong Xeon-E5-2660 dual-CPU machine, the entire analysis pipeline for a small 8200 cells dataset, including bootstrap iterations and computing 2D visualizations, required 2 min and 20 cores, and a maximum of 4.8 GB of RAM. The entire analysis pipeline for a 160K cells’ dataset required 112 min and a maximum of 79-GB RAM on the same machine.

### Evaluating within-MC homogeneity

Following the computation of the MetaCell partition, our pipeline produces diagnostic statistics and plots to evaluate the level of adherence of the metacells to a multinomial sampling model. To visualize large-scale adherence across all genes, we produce per MC plots comparing the coefficient of variation and the fraction of zero counts to the expected under a Poisson model (see examples in Additional file 2: Figure S5). In addition, we visualize adherence to binomial sampling of the top enriched genes per MC by plotting the observed distribution of UMI count and the same distribution sampled from a binomial model (see examples in Fig. 2d). For both observed and expected, counting is done after down-sampling all cells within a metacell to uniform total counts. Finally, global diagnostic matrices over all MCs and marker genes (see example in Fig. 2e) are computed as follows: We down-sample the UMIs to uniform total counts per MC and compute the binomial likelihood of the observed counts, as well as their over-dispersion (observed divided by expected variance). We average these statistics over multiple down-samples and repeat the whole procedure over 999 fake count matrices drawn from the per-MC multinomial model. Per gene and per MC, we compute the empirical *p* value of its likelihood with respect to the binomial null. We output the *p* values and the over-dispersion values and visualize a summarizing heatmap of the latter. Note that when computing binomial statistics, we down-sample with respect to feature and enriched genes only, and that the expected distributions are derived from the pool frequencies constrained to these genes.

### Comparing local approximation accuracy using expression prediction

We designed a cross-validation experiment to quantify how well the MetaCell partition captures local cell-to-cell similarities. We divided the gene set into 100 folds, and leaving out each fold at a time computed cell-to-cell similarities on the remaining genes using four different strategies. We next used these similarities to predict, per cell, the expression level of the left-out genes. Finally, we compared the quality of predictions across all genes. A model that captures accurately local similarities in the expression manifold is expected to produce accurate predictions.

The compared approaches are as follows: (1) predicting using the per-metacell pool frequencies, (2) predicting using the pool frequencies among the top 50 neighbors according to the raw MC similarity matrix *R*, (3) predicting using the pool frequencies of the top 50 neighbors according to Euclidean distances in Seurat’s PCA space, and (4) predicting using the weighted pool frequencies of all cells, where the weights are set as MAGIC’s diffusion similarities (more specifically, MAGIC’s powered Markov affinity matrix). Pool frequencies were computed as regularized geometric means, denoting by *w*_*i*_ the weight of cell *i* in the pool (for strategies 1–3 all weights are 1):

$$ {p}_{g, pool}=\mathrm{e} xp\left[\left(\frac{1}{\Sigma_i{w}_i}{\sum}_{\left\{i\in Pool\right\}}{\mathrm{w}}_{\mathrm{i}}\ \log 2\left(1+7{u}_{gi}\right)\right)-1\right]/\left(\frac{1}{\Sigma_i{w}_i}{\sum}_{\left\{i\in Pool\right\}}{w}_i{u}_i\right) $$

The extent of over-fitting was tested by avoiding the cross-validation design and computing a single similarity matrix using all genes per modeling approach. Regardless of whether cross-validation was used, a cell was never a part of its own prediction pool when comparing prediction accuracy (Fig. 3b, c). In contrast, for plotting the gradients (Fig. 3d, e), the predicted values were generated using all genes and all cells, as in a typical analysis.

Combining Seurat and MetaCell’s filtering criteria, only cells with at least 800 UMIs, number of expressed genes between 800 and 4000, and mitochondrial gene fraction below 0.1 are included. We omitted from the modeling and the evaluation mitochondrial genes and immunoglobulin genes. For MetaCell, we used MC size parameter *K* = 100 and 500 down-samples of 0.75 of the data during the graph resampling stage. For Seurat (package downloaded on 18/3/26), we used gene selection parameters *x*.low.cutoff = 0, *y*.cutoff = 0.8, negative binomial scaling over mitochondrial fraction and number of UMIs, and 40 PCs. For MAGIC (code downloaded on 18/3/19), we used 30 PCs, *k* = 5, ka = 4, epsilon = 1, and *t* = 6.

### Whole organism scRNA-seq analysis

For the *Caenorhabditis elegans* map, we analyzed the whole-organism single-cell dataset published by Cao et al. [42] and generated using methanol-fixed larval L2 stage cells and a split&pool scRNA-seq strategy. We started from a UMI matrix containing 41,449 single cells. We filtered out cells with less than 100 and more than 8000 total UMIs. We used MetaCell to select marker genes with the following criteria: (1) a normalized size correlation below − 0.1 and/or a niche score over 0.1, (2) a minimum of 300 total UMIs observed, and (3) a minimum of 3 UMIs observed in at least three single cells. For MetaCell, we used MC size parameter *K* = 150 and 1000 down-samples of 0.75 of the data during the graph resampling stage. We computed the final partition from the co-occurrence matrix using a size parameter *K* = 30, a minimum MC size parameter of 30 and alpha = 2. We filtered outlier cells using a filtering parameter T_lfc = 4, resulting in a final filtered set of 38,149 cells.

For *Schmidtea mediterranea*, we analyzed the whole-adult single-cell dataset published by Fincher et al. [43] and generated using fresh cells from whole-adult and head area planarian samples and the Drop-seq scRNA-seq technology. We started from a UMI matrix containing 58,328 single cells. We filtered out cells with less than 500 and more than 18,000 total UMIs. We used MetaCell to select marker genes with the following criteria: (1) a normalized size correlation below − 0.1 and/or a niche score over 0.05, (2) a minimum of 300 total UMIs observed, and (3) a minimum of 3 UMIs observed in at least three single cells. In the graph partitioning stage, we used the same parameters as in the *C. elegans* analysis. We filtered outlier cells using a filtering parameter T_lfc = 4.5, resulting in a final filtered set of 56,627 cells.

### Fine clustering using Seurat

Seurat’s clustering algorithm was used for producing a high-resolution clustering of the 160K PBMCs dataset by applying the following procedure: Data was log-normalized and scaled to 10,000 UMIs per cell, 1000 genes with top variance/mean ratio were used as highly variable genes, these genes were rescaled by regressing on per-cell number of UMIs, and PCA reduction to 45 dimensions was applied to the rescaled variable genes. In order to generate a fine clustering solution, we set Seurat’s resolution parameter to 100, using the approximation parameters nn.eps = 0.5 and n.start = 10, which yielded 817 clusters. We note that Seurat is typically executed with much lower resolution values (0.6–3).

## Supplementary information

**Additional file 1:** **Table S1.** Describing key parameters in the MetaCell pipeline and how to tune them. (DOCX 15 kb)

**Additional file 2:** Supplementary figures (Figures S1-S11). (DOCX 9191 kb)

**Additional file 3.** Two strategies for selection of feature genes used in the MetaCell package. (DOCX 14 kb)

## Data Availability

MetaCell’s open-source code is maintained and documented on GitHub [51] and is publicly available under the MIT license from the following Zenodo repository (DOI: 10.5281/zenodo.3334525) [52]. The PBMC data sets were downloaded from the 10x Genomics website [53]. *C. elegans* L2 larva stage dataset was obtained from the Cell Atlas of Worm website [54]. Planaria whole-organism dataset was obtained from NCBI’s GEO [55].
