# Supplementary material for: MetaCell: analysis of single-cell RNA-seq data using K-nn graph partitions
Source: Genome Biol. 2019 Oct 11;20:206. doi: 10.1186/s13059-019-1812-2 (PMC6790056; doi:10.1186/s13059-019-1812-2)
Supplement: Supplementary file 1 — Additional file 1: Table S1. Describing key parameters in the MetaCell pipeline and how to tune them. (DOCX 15 kb) [file 13059_2019_1812_MOESM1_ESM.docx]

| Class | Parameter | Default (Alt.) | Comments/Tuning |
| --- | --- | --- | --- |
| **Features** | T_vm | 0.1 (size cor. Based feature selection) | Minimal normalized variance/mean for selecting genes as features. You can increase this to reduce the number of feature genes, or vice versa. Decreasing should only be used when analyzing very homogeneous datasets (e.g. capturing small variation within a cell line). If your dataset has very high variance in the number of UMIs per cell, feature selection using normalized variance (which inherently relies on data subsampling) is problematic and you should switch to selection using normalized size correlation. |
| **Partition** | Knn | 100 | The number of neighbors the graph balancing procedure is aiming at. Note that in practice, problematic nodes (e.g. outliers) may end up with fewer outgoing edges. We do not recommend modifying this. |
|  | Downsample for raw similarities | F | Should UMI profiles be downsampled before computing raw correlations? This reduces bias for oversampled dataset, but also reduces power and increases noise for datasets with highly variable cell size distribution. |
|  | Knn_core | 30 (20) | The number of most frequency co-clustered neighbors that initiate the co-cluster graph construction. Affects the typical size of metacells in the final solution. Lower this if you are working on a cell-type rich dataset, with many UMIs per cell and relatively low number of cells overall (<10,000). |
|  | min_mc_size | 15 (12) | The minimal size of metacells in the seeding phase. You can lower this if you believe rare cell types are captured efficiently (e.g. within bigger metacells). Do not increase this beyond Knn/5 . |
|  | N_boost | 500 (100) | Can lower N_boost to 100 or 50 to save running time. Effects will be minor for calling “strong” clusters since these are detected robustly on every resampled iteration. |
| **Outliers** | T_lfc | 3 (4) | Defining the log2 value of the outlier threshold on the ratio between observed number of UMIs per gene and the value expected from the metacell multinomial model. Cells with one or more genes showing expression beyond this threshold are defined as outliers.  ***important**: in sparse datasets, or in datasets with significant unexplained variation, this parameter may need to be increased so as to bound the number of called outliers. |
| **Markers** | N_marker | 5 | Number of markers per metacell for generating the standard heatmap. |
| **2D projection** | mc2d_T_edge | 0.05 (0.005) | This parameter determines a threshold on the fraction of cell co-clustering between metacell, which is used when constructing the metacell-metacell graph prior to its 2D projection. For large datasets in which multiple metacells approximate variation of the same transcriptional state, this value should be lowered in order to avoid highly disconnected projection. |
|  | mc2d_max_deg | 3(4) | The target degree of the metacell-metacell graph. This parameter strongly regularizes the graph, but the actual degrees may be higher (since the graph construction process generates asymmetric edges). Consider increasing for large datasets with low cell-type granularity. |

**Table S1: describing key parameters in the MetaCell pipeline and how (if at all) to tune them. All other constants that are discussed in the text do not require user attention.**
