# Supplementary material for: MetaCell: analysis of single-cell RNA-seq data using K-nn graph partitions
Source: Genome Biol. 2019 Oct 11;20:206. doi: 10.1186/s13059-019-1812-2 (PMC6790056; doi:10.1186/s13059-019-1812-2)
Supplement: Supplementary file 2 — Additional file 2: Supplementary figures (Figures S1-S11). (DOCX 9191 kb) [file 13059_2019_1812_MOESM2_ESM.docx]

**
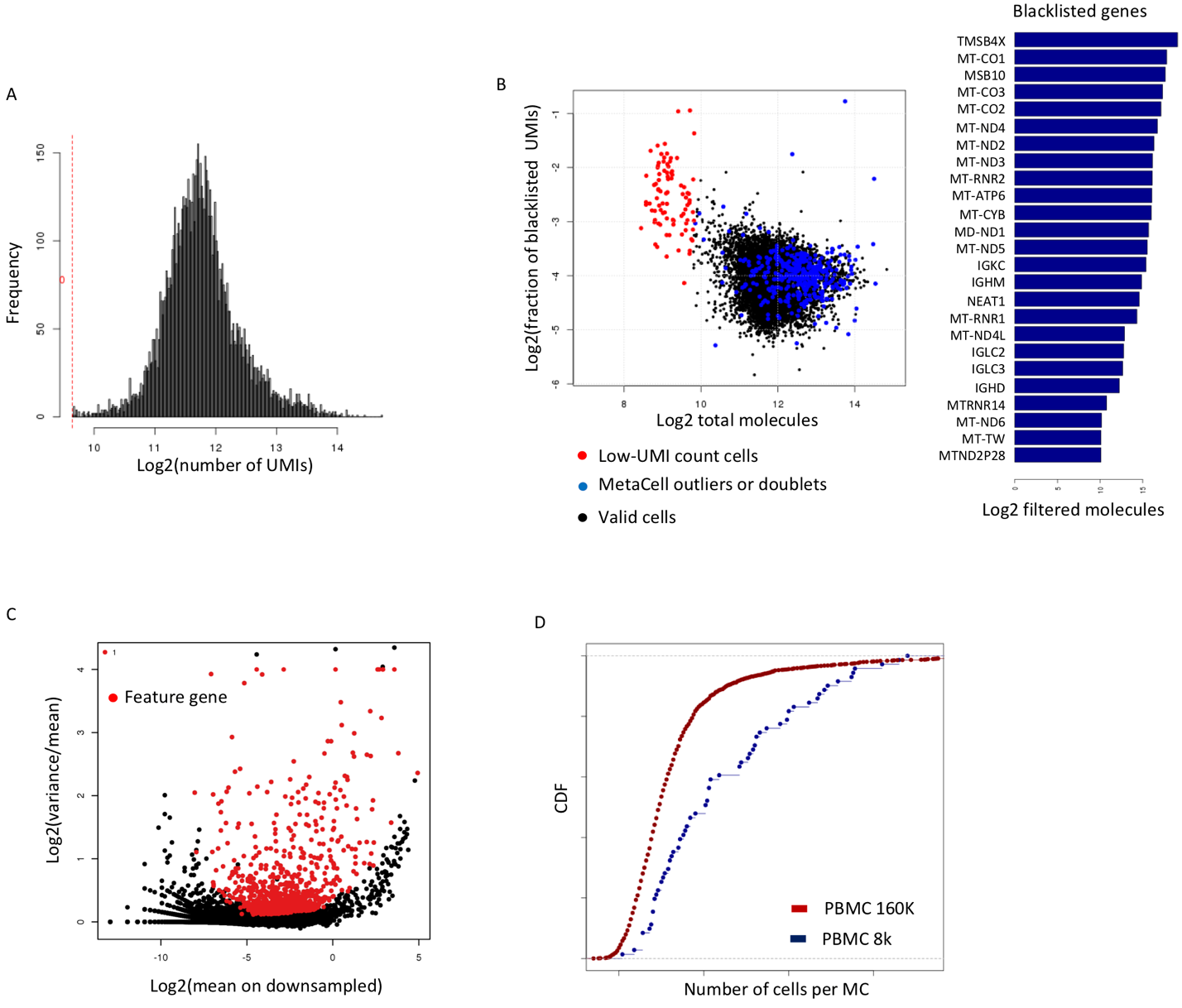
**

**Figure S1:** PBMC filtering and feature gene selection. A) distribution of number of UMIs per cell in the PBMC 8K dataset, after filtering cells with less than 800 UMIs. B) Total number of UMIs (X axis) vs total number of UMIs from genes marked as blacklisted (Y axis). Cells that were filtered based on low UMI count are shown in red. Cells filtered by the MetaCell pipeline based on outlier gene detection or doublet MC annotation are shown in blue. Total number of filtered UMIs for the most abundant filtered genes is shown in bars at the right. C) Gene’s mean UMI count vs normalized variance across the entire PMBC 8K dataset. All statistics are computed on down-sampled UMI matrices. Genes selected as features for computing cell-cell similarity are marked in red. D) Distribution of number of cells per MC (blue). For comparison the distribution of the number of cells per MC is shown for a larger 160K PBMC dataset.

**Figure S2:** Outlier matrix (enlarged version of Fig 1B). Color coded UMI counts for outlier genes (rows) and cells (columns) are shown. Note that genes that define outlier behavior for one cell are frequently valid marker genes for other cells, in particular when outlier cells represent a doublet behavior.

**Figure S3:** Annotating the PBMC 8K MetaCell model using a clustered MC adjacency matrix (color coded heat map, lower part). Identification of subtrees in a hierarchical MC clustering (using a standard Ward R implementation) based on the adjacency matrix (upper panel, subtrees marked in blue, sibling subtree marked in gray), is then followed by detection of enriched genes per subtree. This facilitates the labeling of groups of metacells to specific known or putative biological functions. Labels next to subtree bars indicate the subtree number and its annotation. Note that the granularity of annotation greatly depends on analyst decisions and goals.


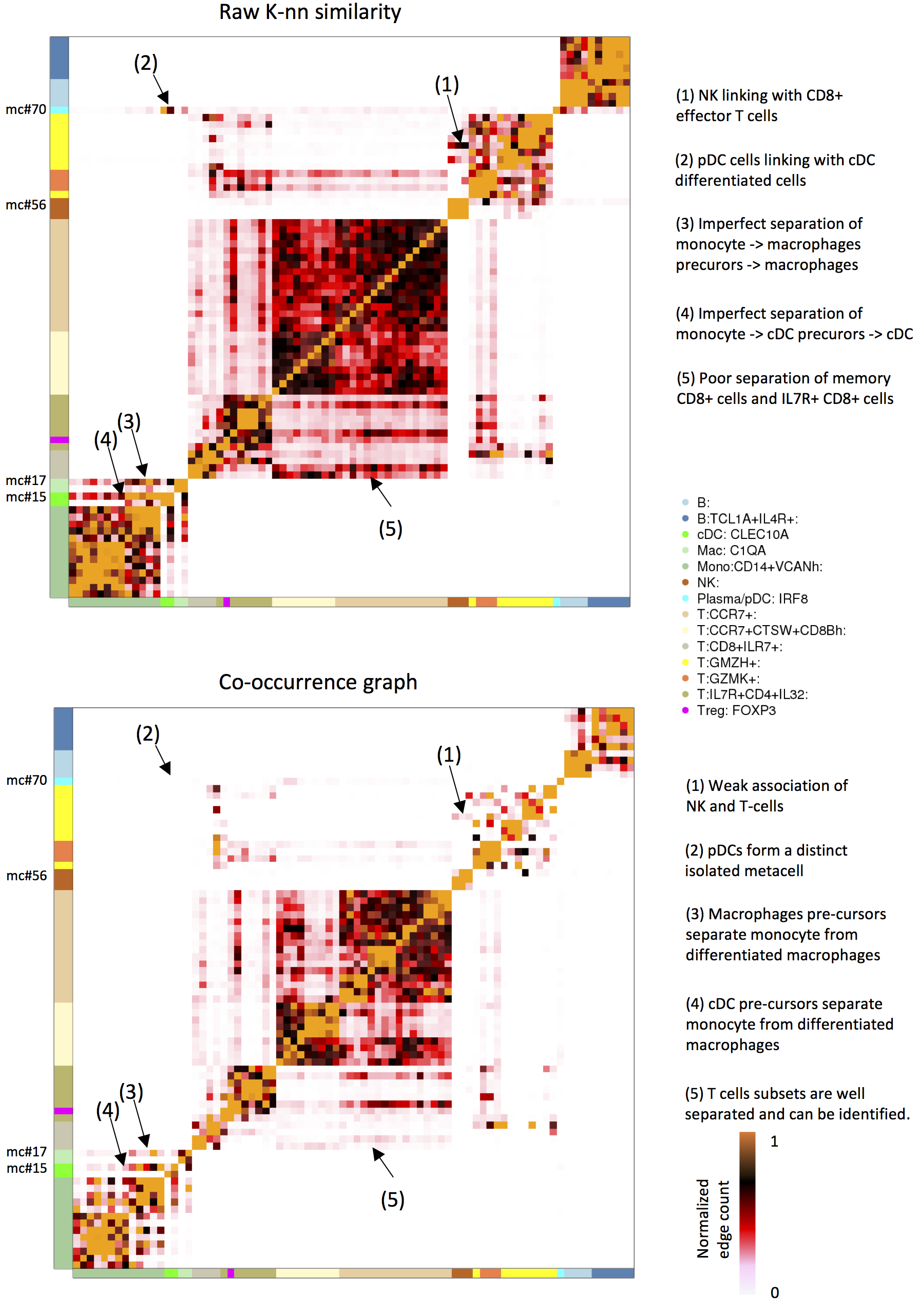


**Figure S4: Changes in MC adjacencies during graph balancing**. Shown are color-coded MC adjacency matrices for the PBMC 8K model using the raw K-nn graph and the co-occurrence graph (matching with Fig 2B). Specific adjacencies are highlighted (numbered 1-5) to exemplify some key effects of the graph balancing procedure.


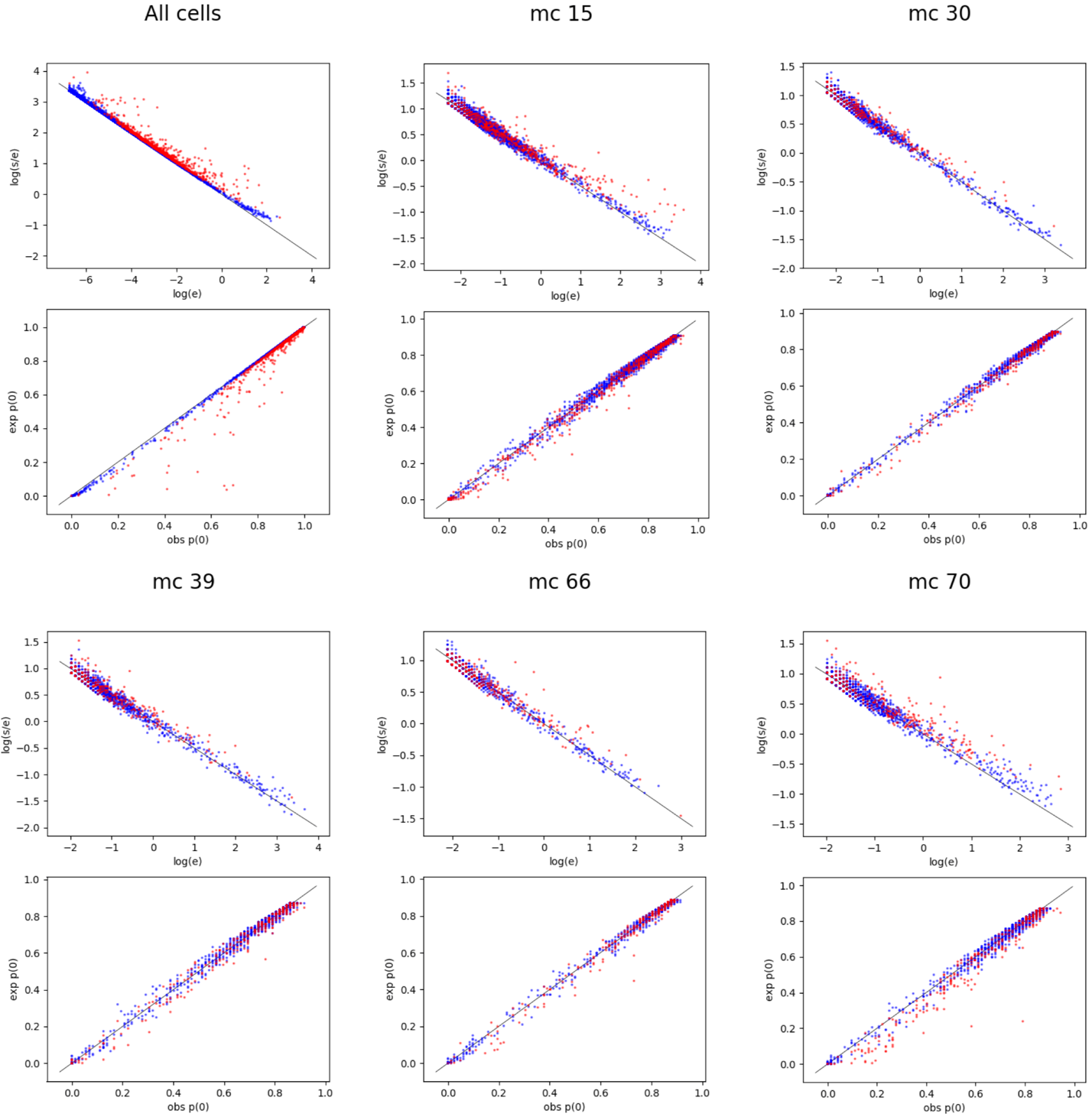


**Figure S5: Poisson plots evaluate over-dispersion within MCs in the 8K PBMC data set.** Per MC, we down-sample all cells to uniform depth, and compute per gene the mean e and standard deviation s of expression values. We plot log(s/e) vs log(e), which should yield a constant -0.5 slope for perfect Poisson approximation. We also plot the expected vs observed fraction of cells with 0 UMIs. Only genes with at least 10 down-sampled UMI’s per MC are shown, and feature genes are colored red. For MCs whose residual variance highly exceeds multinomial sampling variance (see for example MC #70) many genes show over-dispersion and zero inflation.


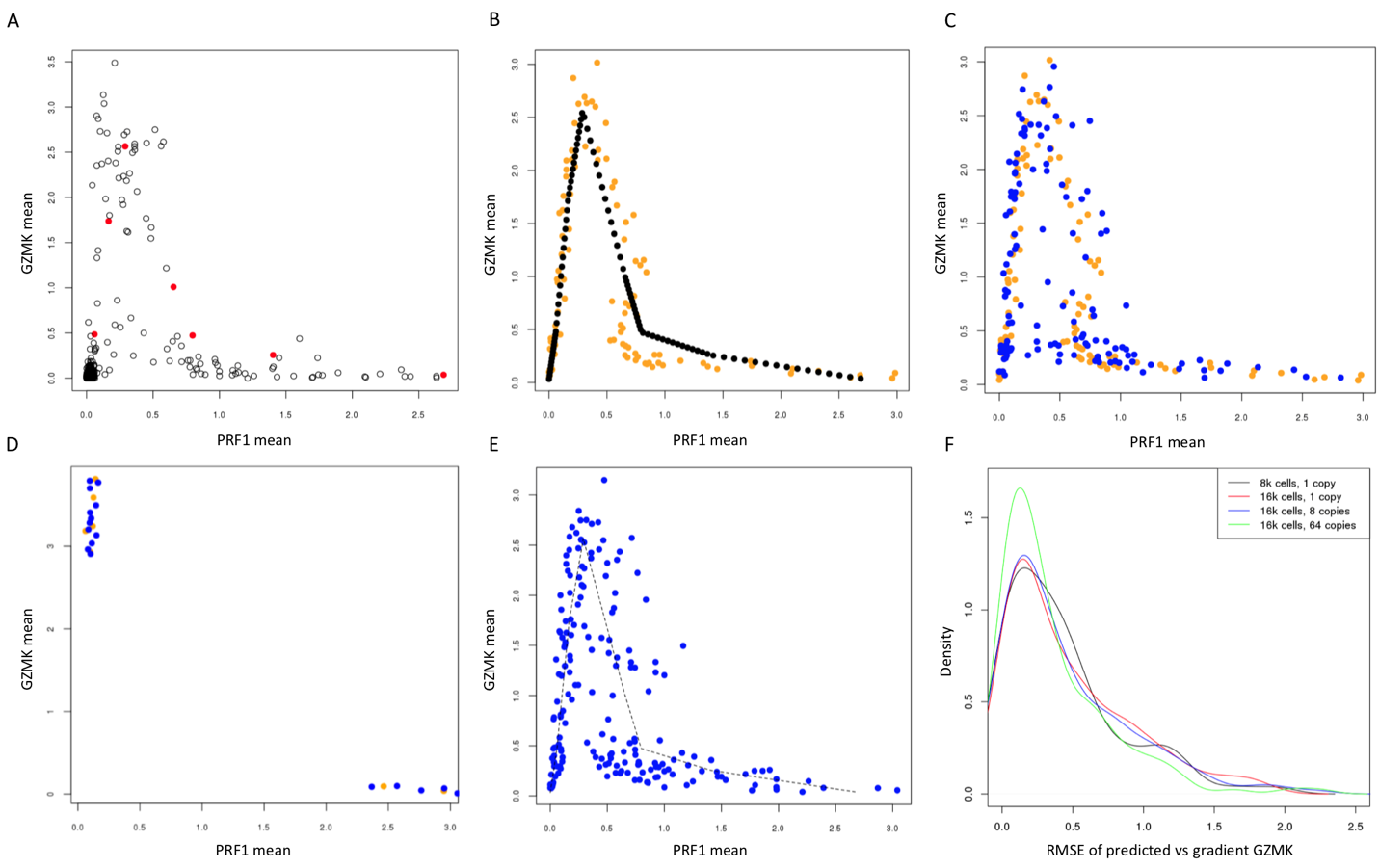


**Figure S6: Testing MetaCell robustness with simulated data.** **A**) Metacells' gene means from real data, capturing a gradient of CD8+ T-cell maturation, where cells exit the naïve state and are transiently expressing a small set of cytotoxic genes (Granzyme K depicted), later upregulating Perforin 1 (depicted) together with multiple cytotoxic genes. Points in red are used for interpolation. See more analysis in Fig 5 – here we used the model only to initialize the simulation. **B**) We generated 100 interpolated metacell parameters along the Perforin-1 simulated gradient (black dots) and drew groups of 100-130  single cell profiles from each model (total of 11768 profiles, with 1400 multinomially sampled UMI’s for each profile). We then inferred multinomial parameters, separately for each group, depicting PRF1 and GZMK estimated frequencies in gold, demonstrating the sampling noise inherent to the scRNA-seq procedure. **C)** Using the same procedures as in B, but now also running the MetaCell algorithm to infer de-novo the grouping. Showing are the metacell inferred parameters in blue, with the parameter inferred from the optimal grouping as a reference in gold. **D)** Here we ran the simulation on only 2 distinct populations, omitting the gradient and confirming that metacells capture distinct states robustly and without smoothing artefacts **E-F)** Here simulation was performed by drawing profiles from a fully quantitative PRF1 gradient (depicted as a dashed line). We compare the accuracy of predicting GZMK given the estimated PRF1 level, when changing the overall number of profiles sampled (16K profiles, red curve), or when extending the simulated gradient with additional copies of the PRF1 and GZMK1 gene (8 copies and 64 copies, blue curve and green curve respectively) – thereby increasing the power to detect the simulated PRF1/GZMK levels.

**Figure S7: Comparison of the *C.elegans* L2 larva MC model versus the reference cell type model.** Top: heatmap showing, for each original cell type in Cao et al. (rows), the distribution of single cells across the metacell (columns). Bottom: heatmap representing the similarity structure between metacells, based on the number of edges in the balanced MC graph that links two cells associated with different MCs. Metacells are color-coded as in Figure 4.

**Figure S8: Comparison of the *S.mediterranea* whole-adult MC model versus the original clustering model.** Top: heatmap showing, for each original cell cluster in Fincher at al. (rows), the distribution of single cells across metacells (columns). Bottom: heatmap representing the similarity structure between metacells, based on the number of edges in the balanced MC graph that links two cells associated with different MCs.


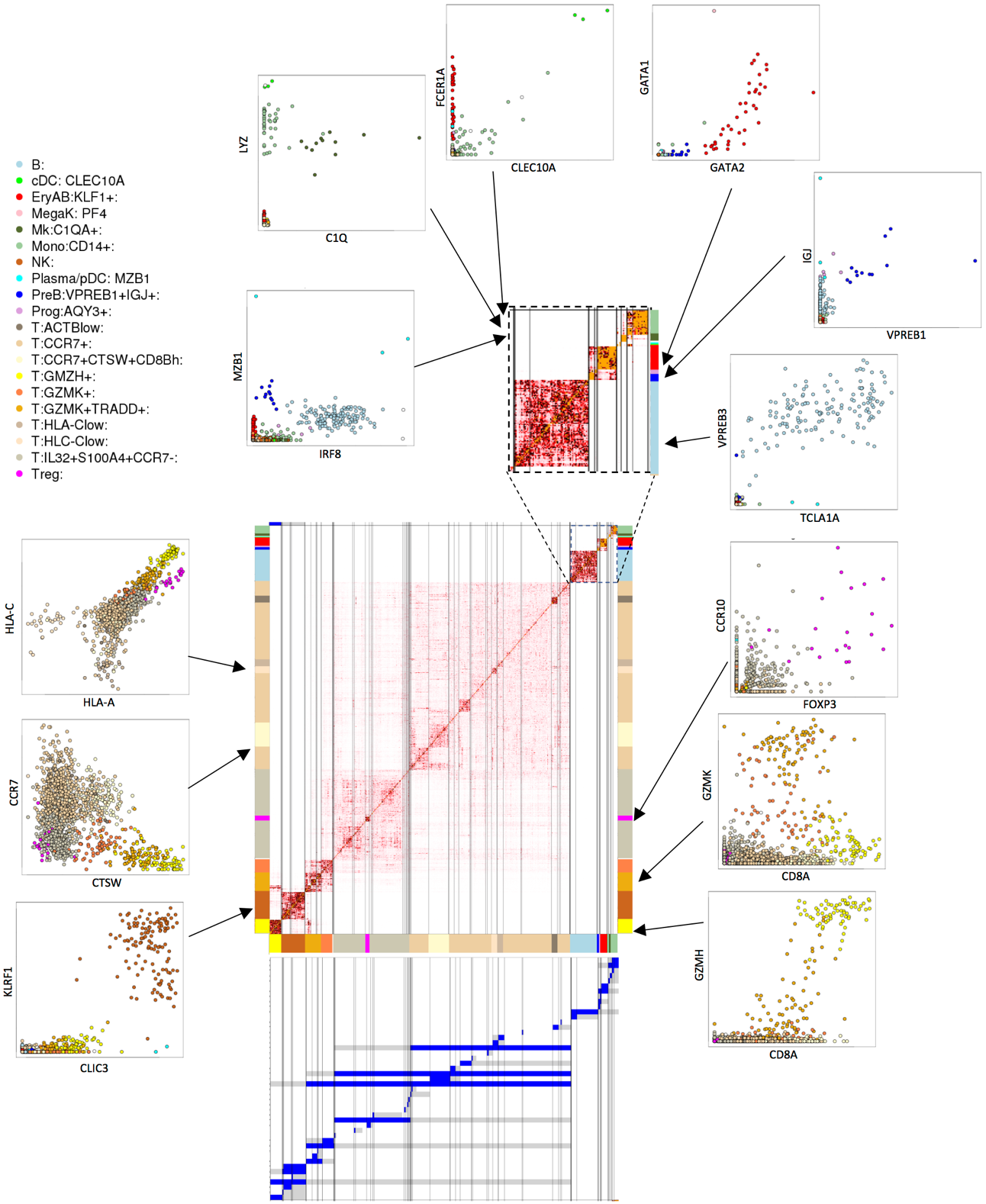


**Figure S9**: Supporting annotation of MC groups in the PBMC 160K model. The MC adjacency matrix (center, color-coded as described above) defines a hierarchy of clustered MCs (depicted as blue/gray bands, bottom: subtrees marked in blue, sibling subtree marked in gray). Specific clusters of MCs are then annotated based on their gene expression signatures. Shown here are select comparisons of gene enrichment (lfp values in X and Y), supporting some key annotations in the PBMC model. Adjacencies between myeloid, progenitors and B cell MCs are enlarged (top right) for clarity.


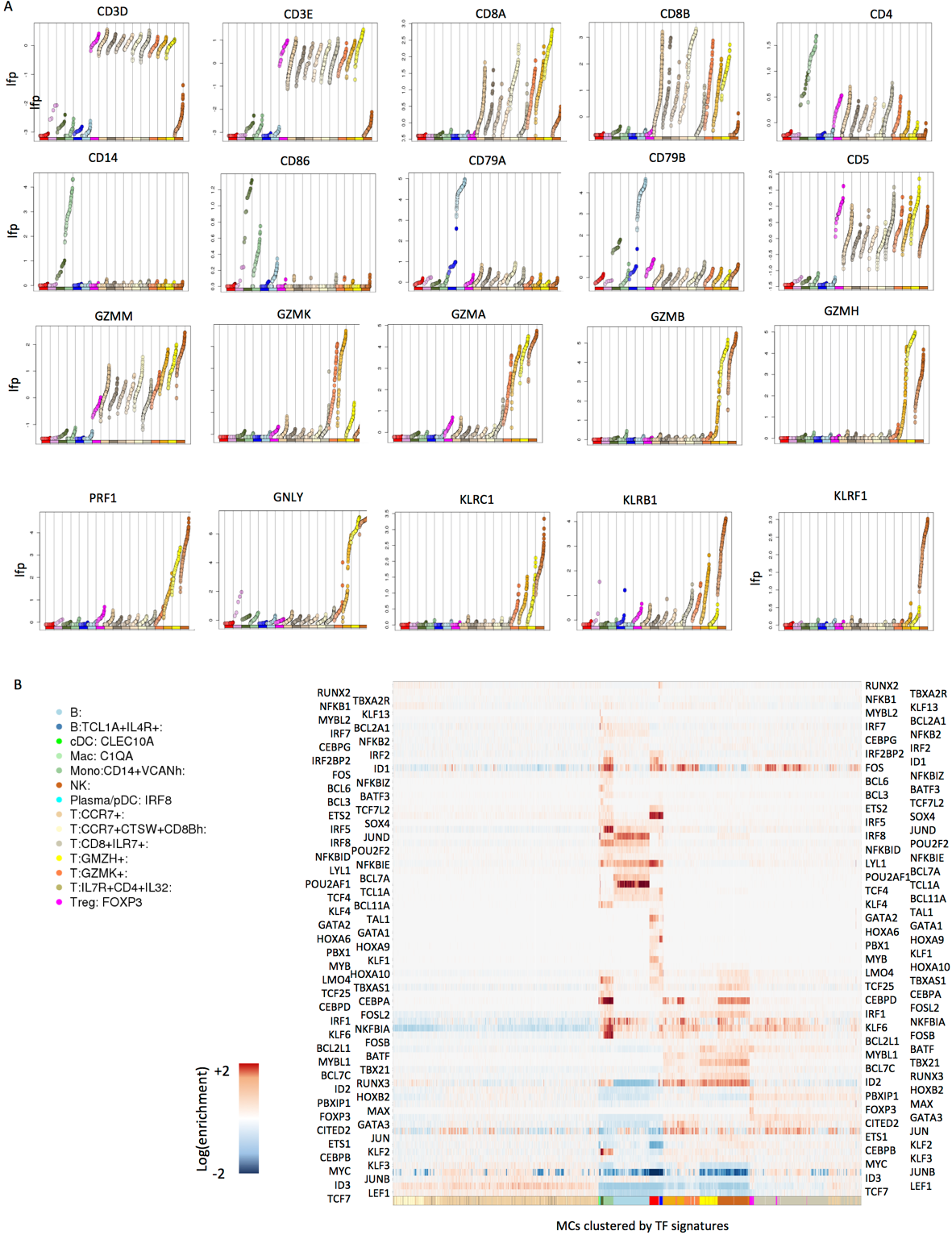


**Figure S10**: **Effector genes are expressed in a convergent fashion. A**) Shown are MC enrichment values for select surface markers and effector genes, ordered according to MC cell type annotation. Note the expression of granzyme and killer genes across multiple subsets of T-cells and NK cells. **B**) Reference mapping of transcription factor expression in the PBMC 160K model. Abundant groups of (mostly naïve) T-cells are defined by relatively few enriched TFs (TCF7, LEF1). Other TFs (e.g. the early-immediate regulators JUN and FOS, or CEBPD) are enriched in multiple cell types. Relatively few TFs are precisely restricted to highly specialized programs (e.g. TCL1A, SOX4).


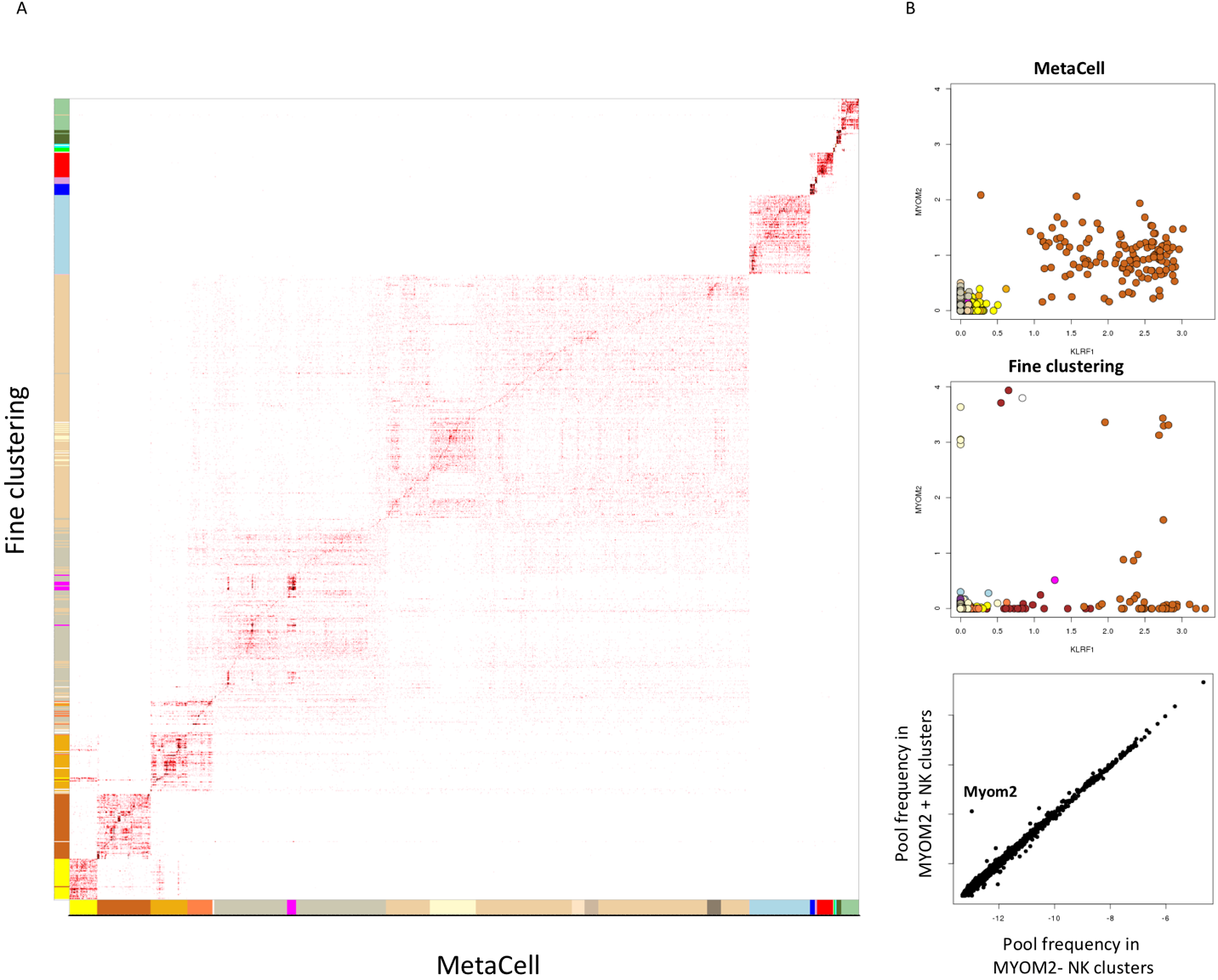


**Figure S11: Comparing MetaCell to fine clustering. A**) The matrix shows the number of cells within each MC (columns) and cluster (rows), where clusters are derived by applying Seurat with parameters forcing high resolution clustering. **B**) Shown are log fold change enrichment per MC (top) and cluster (middle) for KLRF1 (an NK cell marker) and the MYOM2 gene. MYOM2 is generally enriched in NK MCs, but shows highly specific enrichment in specific NK clusters in the fine clustering solution. Bottom: Specific MYOM2 enrichment is suggested to represent overfitting by analysis of overall gene expression in cells within NK clusters with high (Y axis) or low (X axis) MYOM2 levels. Each marker is a gene, and the plot demonstrates no additional genes to be co-variating with MYOM2 within NK cells.
