# Supplementary material for: MetaCell: analysis of single-cell RNA-seq data using K-nn graph partitions
Source: Genome Biol. 2019 Oct 11;20:206. doi: 10.1186/s13059-019-1812-2 (PMC6790056; doi:10.1186/s13059-019-1812-2)
Supplement: Supplementary file 3 — Additional file 3. Two strategies for selection of feature genes used in the MetaCell package. (DOCX 14 kb) [file 13059_2019_1812_MOESM3_ESM.docx]

**Two strategies for selection of feature genes**

Selecting genes $F$ for initiating the modeling of cell-to-cell similarities can be done based on a standard identification of high variance genes, or, in cases where normalizing cells to uniform depth poses a problem, it can be achieved through analysis of the correlation of putative feature genes with cell depth.

To select high variance genes, we define a UMI count threshold $T_{u}= quanile_{i}(u_{i},0.1)$ and create a matrix $W=[w_{gi}]$ by down-sampling $T_{u}$ molecules from each cells for which $u_{i}>T_{u}$, while discarding all other cells. Rows in the matrix W are defined by their mean UMI count, $e_{g}^{ds}$, and by their variance $v_{g}^{ds}$. The variance should be affected by three components. First, since molecules are being sampled from each cell, the sampling variance is expected to be in the order of the mean number of sampled molecules, with a distribution that follows a binomial model (*sampling variance*). Second, RNA concentrations of a gene within homogeneous cell populations are subject to stochastic control that contributes additional variance to our sample (*stochastic variance*). Third, when observing heterogeneous single cell populations, genes that are differentially expressed will be affected by additional variance associated with the sampled sub-populations or cell types (*regulatory variance*). The variance in low expression genes ($e_{g}^{ds}<1$) will be dominated by the sampling component, while for genes with higher variance it is difficult to separate stochastic from regulatory variance. We detect genes whose variance seems more than stochastic as those whose regularized variance $v’_{g} = log2(v_{g}^{ds}/e_{g}^{ds})$is high given their mean expression. Specifically, we compute the empirical trend $v’(e)$ as a function of $e_{g}^{ds}$ using a moving median, and recalibrate the variance over this trend as $v’_{g}^{ds} = v_{g}^{ds} -v’(e_{g}).$ Genes with $\max\left( e_{g}^{ds} \right)>T_{top}, e_{g}^{ds}>T_{e}, v’_{g}^{ds} > T_{vm}$ are selected as features.

Alternatively, when cell depth is highly variable we may prefer to avoid down-sampling the matrix and instead directly correct for the effect of this variation. For each gene $g$, denote by $r_{g}^{sz}= cor\left( u_{gi}, u_{i} \right)$ its Pearson correlation with the cell depth $r_{g}^{sz}= cor(u_{gi}, u_{i}).$ This statistic correlates strongly with mean expression, as increased expression implies lower sampling variance and hence increased correlation with cell depth. For a given level of mean expression this correlation reflects the fraction of sampling variance out of the total expression variance, and truly variable genes will usually show a lower correlation with the cell depth compared to housekeeping genes with similar expression average. We therefore compute an empirical trend $r(u_{g})$ using the median $r_{g}^{sz}$correlation in a moving window of 100 genes ordered by total gene expression $u_{g}$. We then define the normalized depth scaling as $r’_{g} = r_{g}^{sz} - r(u_{g})$. Finally, we select genes with sufficiently high $u_{g}$ and $r’_{g} < T_{gr}$ (typically, $T_{gr} = -0.1$). We note this approach for selecting feature genes may be biased against genes that are enriched for cell types with high $u_{i}$ distribution, and that careful manual analysis of the selected feature genes is recommended in all cases.
